# Multilayer Graphene Sheets and Carbon Nanotubes as Electroconductive Coatings of Glass Fibers for Hierarchical Polymer Composites

**DOI:** 10.3390/polym18212709

**Published:** 2026-11-01

**Authors:** Gabriela Uribe-Riestra, Andrea Chuc, Miguel Rivero-Ayala, Raúl Pech-Pisté, Oscar Solís-Canto, Sombel Diaham, Zarel Valdez-Nava, Alejandro Castillo-Atoche, Francis Avilés

**Affiliations:** 1 Centro de Investigación Científica de Yucatán A.C., Unidad de Materiales, Calle 43 No. 130 × 32 y 34, Chuburná de Hidalgo, Mérida 97205, Yucatán, Mexico; gcuriestra@gmail.com (G.U.-R.);; 2 Univ Toulouse, Toulouse INP, CNRS, LAPLACE, Toulouse, France; 3 Laboratorio Nacional de Nanotecnología, Centro de Investigación en Materiales Avanzados, Av. Miguel de Cervantes 120, Complejo Industrial Chihuahua, Chihuahua 31136, Chihuahua, Mexico; 4 Facultad de Ingeniería, Universidad Autónoma de Yucatán, Avenida Industrias no contaminantes por Anillo Periférico Norte s/n, Mérida 97203, Yucatán, Mexico

**Keywords:** carbon nanostructures, electroconductive coating, air-spraying, damage detection, electrical mapping, adhesion, hierarchical polymer materials

## Abstract

Carbon nanostructures are used to coat engineering fibers to render electrical conductivity to smart polymer composites for applications such as self-sensing, actuation, deicing, and self-healing. Despite their great potential and wide use, specific metrics for assessing quality control of the air-spraying method used to coat the fibers are still missing. Dedicated studies comparing the advantages and disadvantages of using carbon nanotubes or multilayer graphene sheets also remain scarce and controversial. In this study, glass fiber weaves are coated with either multilayer graphene sheets or with carbon nanotubes using the air-spraying method. Key air-spraying parameters such as volumetric flow rate, spot size, and areal density are proposed as quality control metrics and systematically characterized. The resulting coatings were further evaluated for thickness, structural damage assessment through Raman spectroscopy, electrical resistance distribution on the fiber, and adhesion to the fiber surface. The findings indicate that carbon nanotube coatings produced more uniform, more densely packed, and more efficient electroconductive coatings with higher adhesion to the glass fibers at less weight content. They also require less energy density during the dispersion process to produce a uniform coating. In contrast, multilayer graphene sheets required four times more material to achieve coatings with similar or slightly lower electrical resistance distribution. These coatings were integrated into laminated polymer composites to fabricate smart composites with damage self-sensing capabilities. Both materials exhibited high sensitivity to damage detection and localization.

## 1. Introduction

Among many applications in smart polymer composites, carbon nanostructures (CNs) are used to render self-sensing capabilities to hierarchical fiber-reinforced laminated polymer composites. To this aim, CNs have been incorporated into fiber-reinforced polymer composites either by dispersing them within the polymer matrix [1,2,3,4,5] or by coating the fiber surface prior to composite manufacturing [6,7,8,9,10,11,12,13,14,15,16,17,18]. The dispersion of CNs into a polymer matrix has the great disadvantage of increasing resin viscosity and being prone to CN agglomeration due to their high surface area [19,20], which may yield manufacturing problems. Furthermore, the agglomerates can act as stress concentrators, deteriorating the mechanical properties of the composite [19]. On the other hand, methods such as dip coating [8,9,11,15,17,18], electrophoretic deposition [6,7,10,13,16] or in situ growth on fibers [12,14] have been used for several years to incorporate CNs onto the fiber surface. However, their drawbacks and limited scalability to industrial production call for novel, simpler, and industrially scalable methods to coat engineering fibers with CNs, see, e.g., [21,22]. In this sense, the air-spraying technique has emerged as a sound alternative tool with high potential for industrial scaling [20,21]. In this technique, CNs are initially mixed with organic solvents and dispersed by ultrasound [20,21,23]. After ultrasonic dispersion, the CN/solvent solution is spray-coated onto the fiber surface using an airbrush, and then the organic solvent is evaporated. This air-spraying technique has been successfully used to produce fiber-reinforced polymer composites with enhanced interlaminar shear strength [24] and with self-sensing damage capabilities [20,25,26,27,28,29]. In spite of its success, there is no universal consensus on the processing parameters reported for air spraying of these nanostructures, and systematic studies investigating the influence of such parameters on the coating properties are also missing.

Although coating the surface of engineering fibers with CNs has been documented in the literature for both multiwall carbon nanotube (CNT)-coated fibers [8,9,10] and multilayer graphene sheet (GS)-coated fibers [15,16,28] for damage sensing applications, very few studies have made a direct comparison of the coatings features using CNs of different morphologies [13,17,18,30]. In this regard, Hao et al. [13] coated glass fibers (GFs) with CNTs and GSs using an electrophoretic method. The coated fibers were incorporated into fiber-reinforced polymer composites to make the composites sensitive to strain and damage. GS-coated fibers showed higher sensitivity to mechanical strain and damage, which was associated with the layered structure of the coating on the GF surface.

On the other hand, Bhandari et al. [30] studied the electrical conductivity and electrical sensitivity of hierarchical composites modified with CNTs and hybrids of CNTs and GSs. The authors observed that composites modified solely with CNTs exhibited the highest electrical conductivity. Hierarchical composites modified with a combination of CNTs and GSs presented the highest electrical sensitivity to strain. It is thus clear that conductivity and electrical sensitivity to strain and damage of CN-coated fibers depend on the nanostructure morphology, its dimensions and physicochemical properties. However, further studies are needed to establish a consensus regarding the relative advantages and limitations of one over the other. Moreover, efforts towards international standardization demand a conspicuous definition of a set of quantifiable processing parameters for the air-spraying method.

To address this gap, this work presents detailed characterization and a comparative study of glass fiber surface coatings using two dissimilar carbon nanostructures, viz., CNTs and GSs. The study focuses on the spray-coating processing parameters, distribution and dispersion on the fiber, adhesion, and the resulting electrical resistance to produce hierarchical composites, which are electrically sensitive to damage. Regarding the spray-coating processing parameters, the volumetric flow rate, areal density, spot size of the air gun, and coating thickness are proposed as quality-control parameters towards quantitative standardization and compared for both nanostructures. The characterized parameters proposed are key features for quality control and process standardization of the air-spraying method for coating fibers with carbon nanostructures, especially for their incorporation into polymer composites.

## 2. Materials and Methods

### 2.1. Materials

E-type glass fibers in a 0°/90° balanced weave architecture (GFWs), sourced from Poliformas Plásticas (Mérida, Mexico), were used as the substrate, coated with one of the two commercial CNs used herein. The GFWs have an areal density of 600 g/m^2^ and a tow width of ~5 mm, where the individual fibers have an average diameter of 15 μm and a density of 2.54 g/cm^3^ [31]. Commercial GFWs were supplied with a proprietary sizing formulation comprising an organic film former, silane coupling agents, surfactants, and lubricants. Previous physicochemical analyses of the same fibers used in this work revealed the presence of epoxy prepolymer as the film former in the sizing and an organic silane coupling agent whose structure is similar to 3-(trimethoxysilyl)propyl methacrylate [19].

The GFW was coated either with CNTs or GSs and comparatively characterized. The CNTs, acquired from Cheaptubes Inc. (Grafton, MA, USA), had a mean length (*L_CNT_*) of 2.6 µm, a mean outer diameter (*d_CNT_*) of 29.1 nm, and purity > 95%. The GSs, sourced from XG Sciences (Michigan, IN, USA), were type “H25” with a mean lateral size (*d_GS_*) of 20.9 µm, a thickness ranging from 3.8 to 6.1 nm, and 11 to 18 layers [32]. Ethanol (purity > 96%) was chosen as the solvent for CN solution air-spraying over the GFW to prevent unintended removal of the fiber surface coating (sizing).

As an application for polymer composites, the fibers, once coated with CNs, were further used for fabrication of hierarchical laminated polymer composites through resin infusion. To this aim, a Dramane Momentum 470-300 vinyl ester resin (VER, Ashland Global, Covington, KY, USA), was used as the polymer matrix. Cobalt naphthenate (CoNap, 0.5 wt.%) and methyl ethyl ketone peroxide (MEKP, 0.5 wt.%) were used as promoter and initiator of the crosslinking polymerization of VER. Both reagents were acquired from Plastiformas de México (Mexico City, Mexico).

### 2.2. Air-Spraying Method for Coating Glass Fiber Weaves with Carbon Nanostructures

The air-spraying method was chosen to cover the surface of GFWs because of its versatility and scalability. For this, the CNs (either CNTs or GSs) were initially ultrasonically dispersed in ethanol to obtain a CN solution. The concentration of the solution and spraying conditions were first investigated to produce CN-coated GFWs with electrical resistances in the kΩ range, which is a range suitable for self-sensing applications. This required an initial iterative study of different concentrations of CNs in solution for the CNTs and GSs (not detailed herein). The final selected concentrations which yielded similar values of electrical resistances were 1 mg/mL for CNTs and 4 mg/mL for GSs. This is because at 1 mg/mL, the fibers coated with GSs were still electrically insulating. Initial tests also proved that GSs demand significantly higher ultrasonic energy density for adequate dispersion than CNTs, so the experimental conditions were adjusted accordingly. For the CNT-coated GFWs, a 1 mg/mL solution was subject to ultrasonic dispersion in bath (70 W, 40 kHz) for 30 min to limit fragmentation. This yielded an estimated upper bound energy density of 6.3 × 10^7^ J/m^3^. For the GS-coated GFWs, a higher concentration of GSs (4 mg/mL) was used and dispersed using higher ultrasonic energy. Initial experiments showed that dispersing GSs with the same ultrasonic energy density as for CNTs (either at 1 mg/mL or 4 mg/mL) rendered an overly agglomerated, heterogeneous, and highly non-uniform coating over the GFWs. This coating is not functional for this application since it produced erratic electrical measurements that prohibited practical applications of the fibers as self-sensing materials. Therefore, the final 4 mg/mL GS/ethanol solution described herein was dispersed with an ultrasonic horn (225 W, 70 kHz) for 30 cumulative min, using alternating 30 s on/off cycles, yielding and energy density of 6.8 × 10^9^ J/m^3^. Therefore, the CNTs and GSs are not compared under identical processing conditions, but under two optimized processing routes that yield functional coated fibers with similar electrical conductivity. The resulting CN/ethanol solution was poured into the cup of an air gun with a 1.4 mm flat-fan nozzle, which was connected to an air compressor at 1.03 bar (15 psi). The air gun was then mounted on the fixture of a 3-axis computerized numerical control (CNC) engraving machine, positioned 10 cm above the GFW, which is held at the mobile support plate. The planar dimensions of the coated GFW were 160 mm × 100 mm. Fiber coating was achieved by triggering the air gun while the CNC engraving machine moved the support plate in a continuous serpentine raster path at a constant rate of 0.9 m/min, consisting of alternating upward and downward passes connected by lateral shifts to the right of 20 mm. After the CNs were deposited, the CN-coated GFW was dried in a convection oven at 70 °C for 4 h to remove residual solvent.

### 2.3. Characterization of Air-Gun Parameters for the Deposition of Carbon Nanostructures

**Figure 1:**
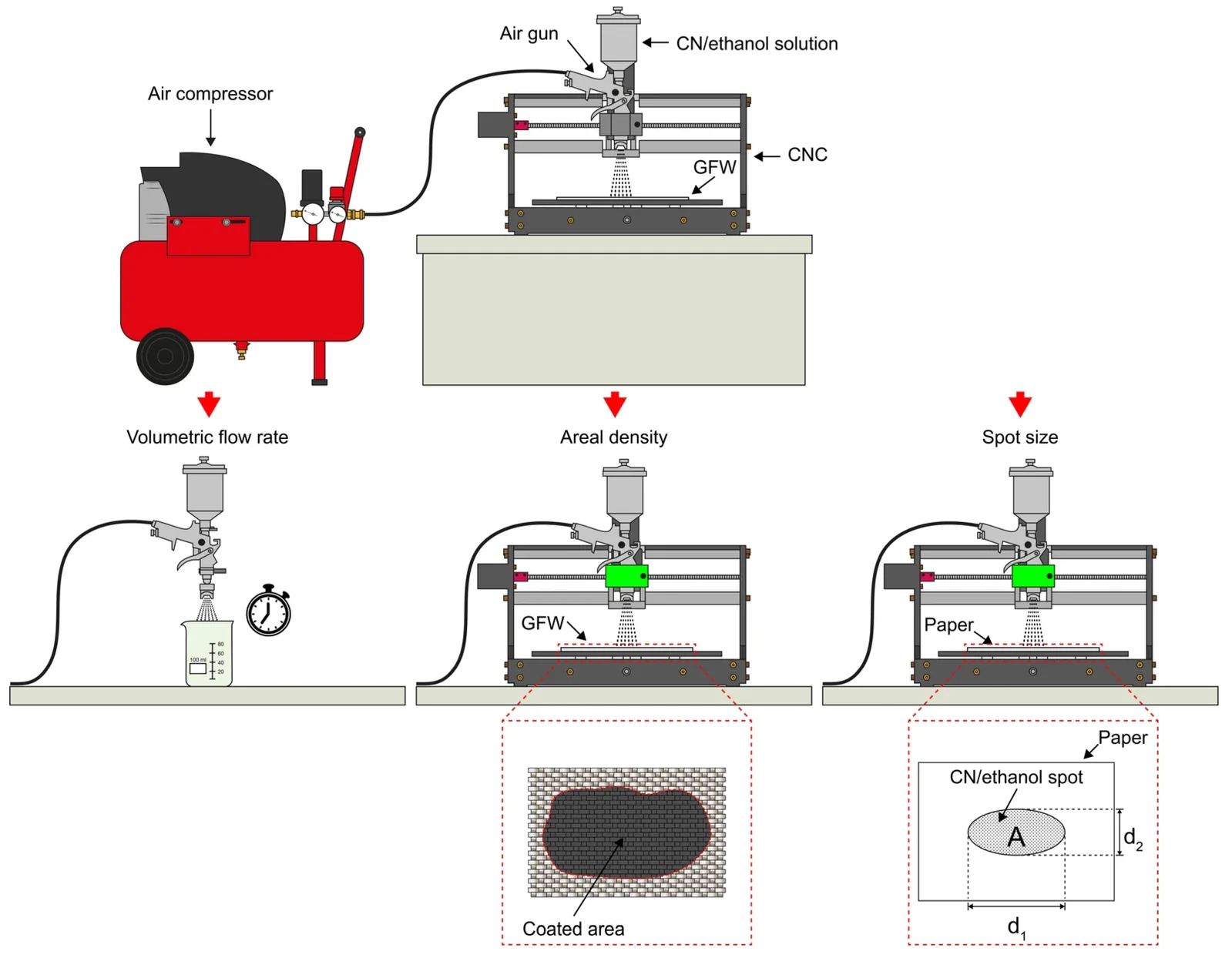
Schematic of experimental setup used for characterization of air-gun deposit parameters.

The deposition of CNs over substrates such as fibers using an air gun is strongly dependent on the experimental parameters used. However, this is frequently not reported in the literature, which hinders proper construction of universal structure–property correlations. In order to provide quantitative parameters of the experimental conditions used herein, three parameters of the air-gun coating process are reported. A schematic representation of the experimental setup used for determination of the volumetric flow rate (Section 2.3.1), areal density (Section 2.3.2) and spot size (Section 2.3.3) is presented in Figure 1. To ensure reproducibility of results, all characterizations of the coatings were conducted in triplicate.

#### 2.3.1. Determination of Volumetric Liquid Flow

The volumetric flow rate at the outlet of the air gun used for fiber coating was characterized. For this, a known volume (*V*) of CN/ethanol solution was poured into the cup of the air gun. The air gun was connected to an air compressor at 1.03 bar (15 psi), and the solution was fully expelled from the air gun and collected into a beaker. The elapsed time required to fully expel three volumes of fluid (30 mL, 60 mL, and 90 mL) was measured in separate experiments. A plot of V vs. t was generated from these results, and the data were fit to a linear function. The volumetric flow rate was determined as the slope of this fit. For statistical reproducibility, each volume was tested three times. This procedure was applied for both CNTs and GSs.

#### 2.3.2. Measurement of Air-Spray Spot Size

A dedicated experiment was conducted to estimate the spot size area of a single air-gun spray. For this, it was assumed that CNs in solution form a cone between the nozzle of the air gun and the GFWs, resulting in an elliptical spot at the base of the cone (substrate surface). To better visualize this spot, a sheet of white printing paper (21.6 cm × 27.9 cm size, 75 g/m^2^ density) was placed as substrate instead of the GFWs. To produce the spot, the air gun was filled with 30 mL of the CN/ethanol solution and mounted on the CNC engraving machine. Then, the air gun was triggered for 5 s to produce a well-defined black spot on a white paper sheet placed on the mobile support of the engraving machine. Photographs of the CN/ethanol solution spots were taken using a 48 Mpx Samsung GW1 CCD photographic camera. Spot area (*A*) and its major (*d*_1_) and minor axes (*d*_2_) were measured using image-processing software (ImageJ, version 1.54, RRID:SCR_003070, National Institutes of Health, Bethesda, MD, USA). This process was conducted six times for each CN to ensure statistical significance and to obtain mean values and standard deviation.

#### 2.3.3. Measurement of Areal Density

Areal density, defined as CN mass per unit area covering the GFW, was another parameter used to characterize the fiber coating process. To estimate the areal density, a known mass of CNs in solution was deposited on a 350 mm × 350 mm GFW in one pass. The dimensions used for the GFW in this experiment are intentionally too large, so the CN solution is fully depleted without covering the full GFW area. Three masses (*m*) of CNs, viz. 10 mg, 30 mg, and 70 mg, were dispersed in ethanol at a concentration of 1 mg/mL for CNTs and 4 mg/mL for GSs. Each measurement was performed three times for each CN mass. The solution was then deposited on the GFW using an air gun mounted in a CNC engraving machine at a constant speed of 0.9 m/min. The GFW area comprising coated and uncoated sections was photographed and analyzed using the image processing software ImageJ to measure the covered area. Areal density was calculated as the slope of the linear fit of the measured area data versus the known masses, with the intercept fixed at (0,0). The uncertainty of the experiment includes potential unaccounted small mass losses, such as a small amount of CN particles remaining on the walls of the container after air-gun depletion. Therefore, the reported values represented an upper bound.

### 2.4. Characterization of Carbon Nanostructure-Coated Glass Fibers

The glass fibers coated with CNs were characterized using scanning electron microscopy (SEM), atomic force microscopy (AFM), Raman spectroscopy, peeling adhesion tests and electrical resistance spatial maps, as described below.

#### 2.4.1. Morphology and Thickness Characterization of CN-Modified Fibers

The dispersion and distribution of CNs over GFWs were examined by both AFM and SEM imaging. SEM images were obtained with a JEOL JSM 6360-LV microscope(Tokyo, Japan), featuring magnifications ranging from 100× to 20,000×. SEM images were acquired from 1 cm-long tows extracted from the CN-coated GFW. The GF samples were sputtered with a thin (~4 nm-thick) gold layer.

AFM topographical images of the CNT-coated fibers were acquired using an SPM-8 microscope (Bruker, Kontic, Belgium) equipped with a silicon tip (RTESPA-300) with a resonant frequency of 320 kHz and spring constant of 40 N/m. Square AFM images with an area of 3 μm × 3 μm were used to quantify the roughness of the coating using the proprietary NanoScope Analysis software(version v150r3). For that, 4 images of each CN were divided into six square sections, and the root mean square roughness was calculated in each section, excluding the image borders. The high surface roughness and height variations of the GS-coated fibers exceeded the AFM tip/scanner *z*-axis dynamic range, and thus AFM images reported include only those of the CNT-coated fibers.

To estimate the coating thickness, cross-sections of individual fibers were examined with a high-resolution SEM. For that, CN-coated fibers were immersed in liquid nitrogen and subsequently fragmented. The fragmented CN-coated GFWs were examined using SU-3500 Hitachi SEM equipment (Hitachi High-Tech, Tokyo, Japan), whose resolution can reach up to 3 nm. Microscopy images were acquired at magnifications between 4200× to 21,000×. The thickness of the coating was estimated from these images using the image processing software.

#### 2.4.2. Raman Spectroscopy of Coated Glass Fiber Weaves

To further assess the potential fragmentation and degradation of structural properties of the CNs after ultrasonic dispersion, Raman spectroscopy was conducted on both as-received CNs (i.e., before fiber coating) and on CNs coating the GFWs. This analysis was conducted using a Renishaw InVia confocal Raman spectrometer (Gloucester, UK), with a 532 nm (red) laser, 50× objective lens and 60 s exposure time. Raman bands were fit to Gaussian functions to obtain the center positions, intensity, and full-widths-at-half-maximum. Three specimens of as-received CNs (before ultrasonic dispersion) and three of CN-coated GFWs (after ultrasonic dispersion) were analyzed.

#### 2.4.3. Peeling Tests

**Figure 2:**
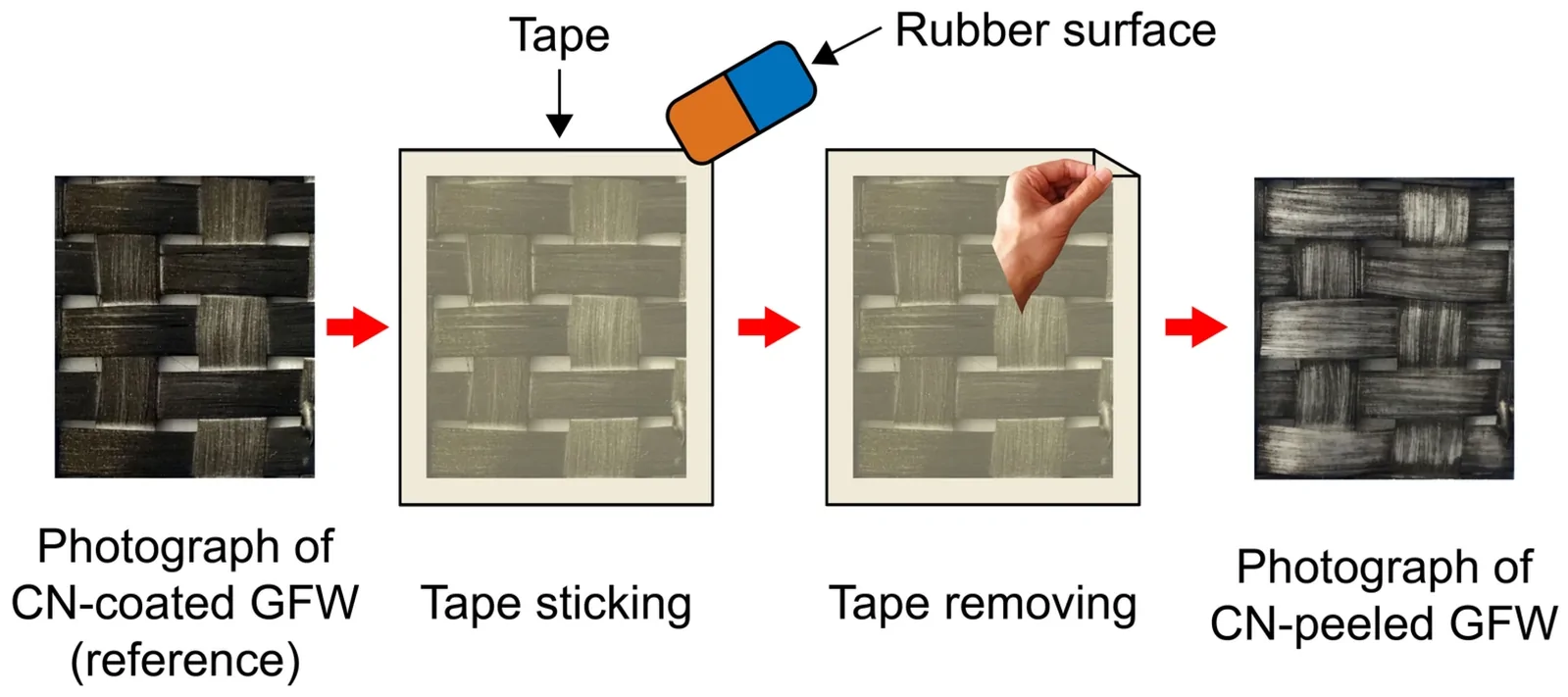
The schematic of the peeling test process.

The relative adhesion of CNs to the surface of GFWs was quantified using a modified tape-peeling test inspired in ASTM D3359 [33]. To this aim, CN-coated GFW specimens were sectioned into 20 mm × 20 mm squares. Masking tape (3 M, Maplewood, MN, USA) was gently applied to each section with uniform pressure using a smooth surface and immediately removed, taking part of the CNs with the sticky surface of the tape and leaving a remnant CN coating on the GFW surface. Photographs of the GFW before (reference image) and after tape removal were taken using a 16 Mpx OV16A19 CMOS camera (OMNIVISION, Santa Clara, CA, USA) in close-up mode. The overall procedure of the peeling test is depicted in Figure 2.

Photographs of the coating were taken before and after each of the three peelings for three samples. The remnant area covered by CNs was quantified using a dedicated pixel counter software. The in-house software binarizes the reference image (before peeling) and those taken after each peeling and counts the pixels exceeding a user-defined threshold to distinguish between covered and uncovered pixels. The remaining dark pixels represent the remaining CN area left on the GFW after peeling. The ratio between this remnant CN-covered area and that of the reference image (before peeling) was reported as a relative metric of interfacial adhesion, named herein the “remaining covered area”. Three replicates of different batches were evaluated for each nanostructure coating.

#### 2.4.4. Electrical Resistance Mapping of CN-Coated Fibers

**Figure 3:**
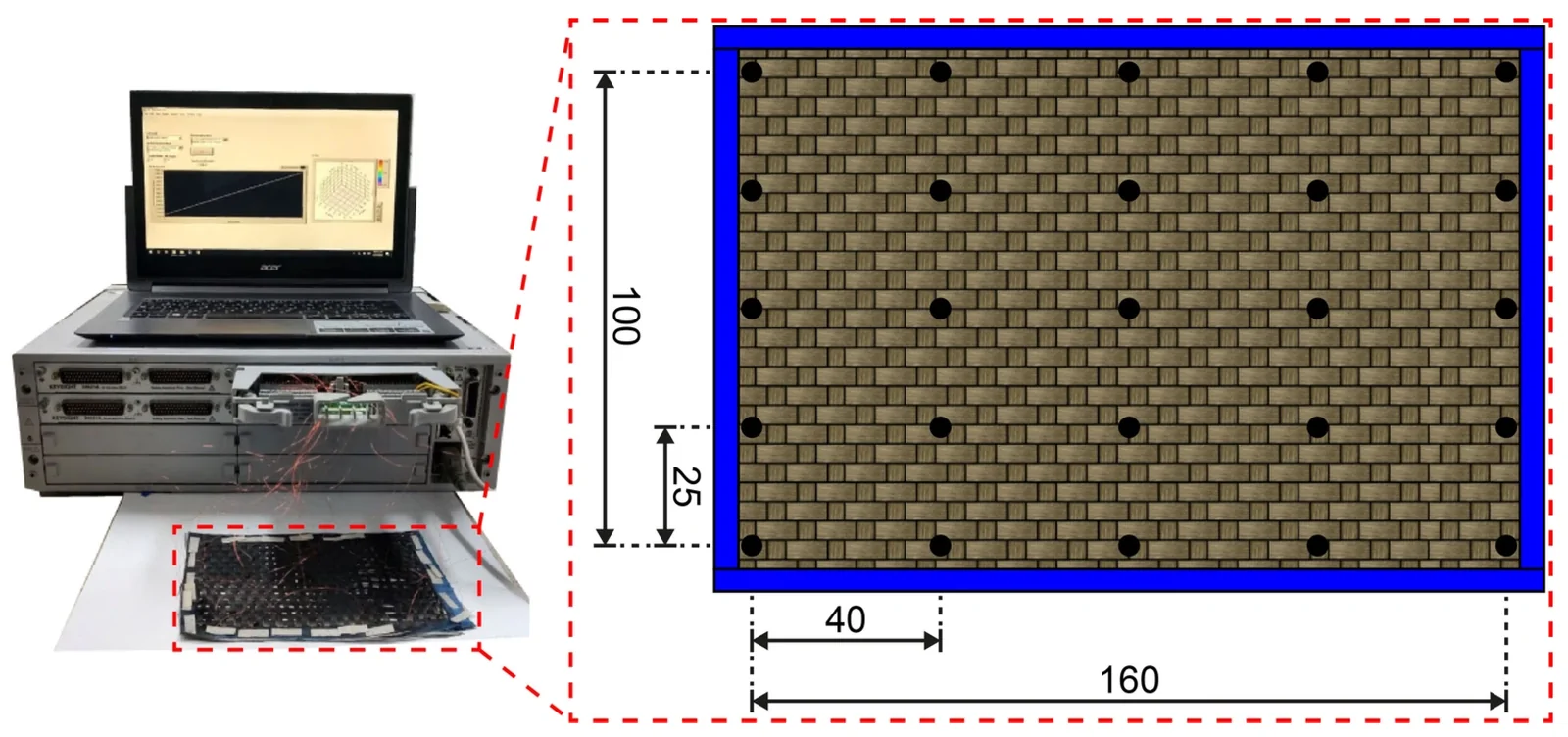
Experimental setup and electrode distribution used for the electrical resistance measurements. Black dots indicate electrode positions. Dimensions in mm.

The macroscopic homogeneity of the CN coating was evaluated by mapping the distribution of electrical resistance across the surface area of the GFWs. These electrical resistance maps also serve as a quality control tool for the prospective application of the CN-coated fibers as smart materials, as will be further discussed. To this aim, a grid of 25 electrodes, arranged in 5 rows and 5 columns, were attached to the surface of a 160 mm × 100 mm GFW. Copper wires serving as electrodes were affixed to the GFW surface using a droplet of carbon/water-based “Bare Conductive” electroconductive paint (London, UK). Electrical resistance of the 25 electrodes was measured with a Keysight 34980A multifunction switch/measure system (Santa Rosa, CA, USA), equipped with an 80-single-relay module (34923A). Measurements were acquired between each electrode and its immediate neighbors, resulting in a total of 72 measurements, i.e., 20 horizontal measurements (*x*-direction), 20 vertical measurements (*y*-direction), and 32 diagonal measurements. Each of the 72 electrical resistance data points was assigned to an (*x*,*y*) coordinate at the midpoint between the two electrodes. For diagonal measurements, the value assigned to a given (*x*,*y*) location was calculated as the average of the two intersecting electrical resistance measurements. The data were visualized as a continuous isocontour map using a MATLAB2015b (Natick, MA, USA) script that applied cubic spline interpolation between discrete points. The experimental setup used for the electrical resistance measurements as well as the distribution of electrodes used for acquiring the electrical resistance is depicted in Figure 3. Three specimens were tested showing similar trends, and a representative one for each group is shown in the Results Section.

### 2.5. Manufacturing of Laminated Composites

Laminated composites were fabricated using vacuum assisted resin transfer molding. Fifteen 18 cm × 18 cm GFWs were used as preforms, being only one (the top one) coated with CNs to create an electroconductive sensing layer. A high-concentration CNT/VER mixture was used as conductive paste, enabling electrode attachment without embedded wires. The paste consisted of 1.0 wt.% CNTs dispersed in VER using an ultrasonic horn operating at 225 W and 70 kHz. The CNT/VER paste was applied as 5 mm thin lines covering the whole length (18 cm) of the CN-coated GFW surface, spaced 25 mm apart. This allowed for the formation of a small conductive region at the side-edge of each beam specimen after cutting the manufactured panels into 25 mm wide beam specimens. After sealing the mold, VER mixed with 0.5 wt.% promoter (CoNap) and 0.5 wt.% initiator (MEKP) was infused at 20 psi. Vacuum was maintained until the VER fully impregnated the GFWs, and air bubbles were removed. The laminated composite was cured at room temperature for 24 h and post-cured at 120 °C for 4 h. Six laminated beam specimens, each measuring 154 mm × 25 mm × 7 mm, were sectioned using a diamond saw, in accordance with the ASTM D7264 standard [34].

### 2.6. Electromechanical Bending Tests of Laminated Composites

Laminated composite specimens were tested under four-point bending while simultaneously measuring their electrical resistance using the 42 electrodes described above, to allow the construction of electrical resistance maps. Electromechanical bending tests were conducted under monotonic loading until failure using a Shimadzu AG-I universal testing machine (Kyoto, Japan), with a displacement-controlled rate of 1 mm/min. A span length of 112 mm was used, resulting in a span-to-thickness ratio of 1:16. Load introduction elements were centered and spaced 56 mm apart. Full field strains were simultaneously obtained during the mechanical tests using the digital image correlation (DIC) technique. DIC was conducted using an ARAMIS 5M LT system, equipped with two 5-megapixel cameras with 35 mm lenses, capturing images at a rate of 1 image/s. Image processing was conducted with the ARAMIS V6.3.1 software, employing subsets of 19 × 19 pixels and a step size of 15 pixels. The set of 42 electrodes was placed at the top surface to acquire electrical resistance signals during the electromechanical bending tests. A more detailed description of the electrode distribution, electrical resistance data acquisition, and the generation of isocontour maps for the fractional change of electrical resistance (Δ*R*/*R*_0_) can be found in [8].

## 3. Results

### 3.1. Volumetric Flow Rate

**Figure 4:**
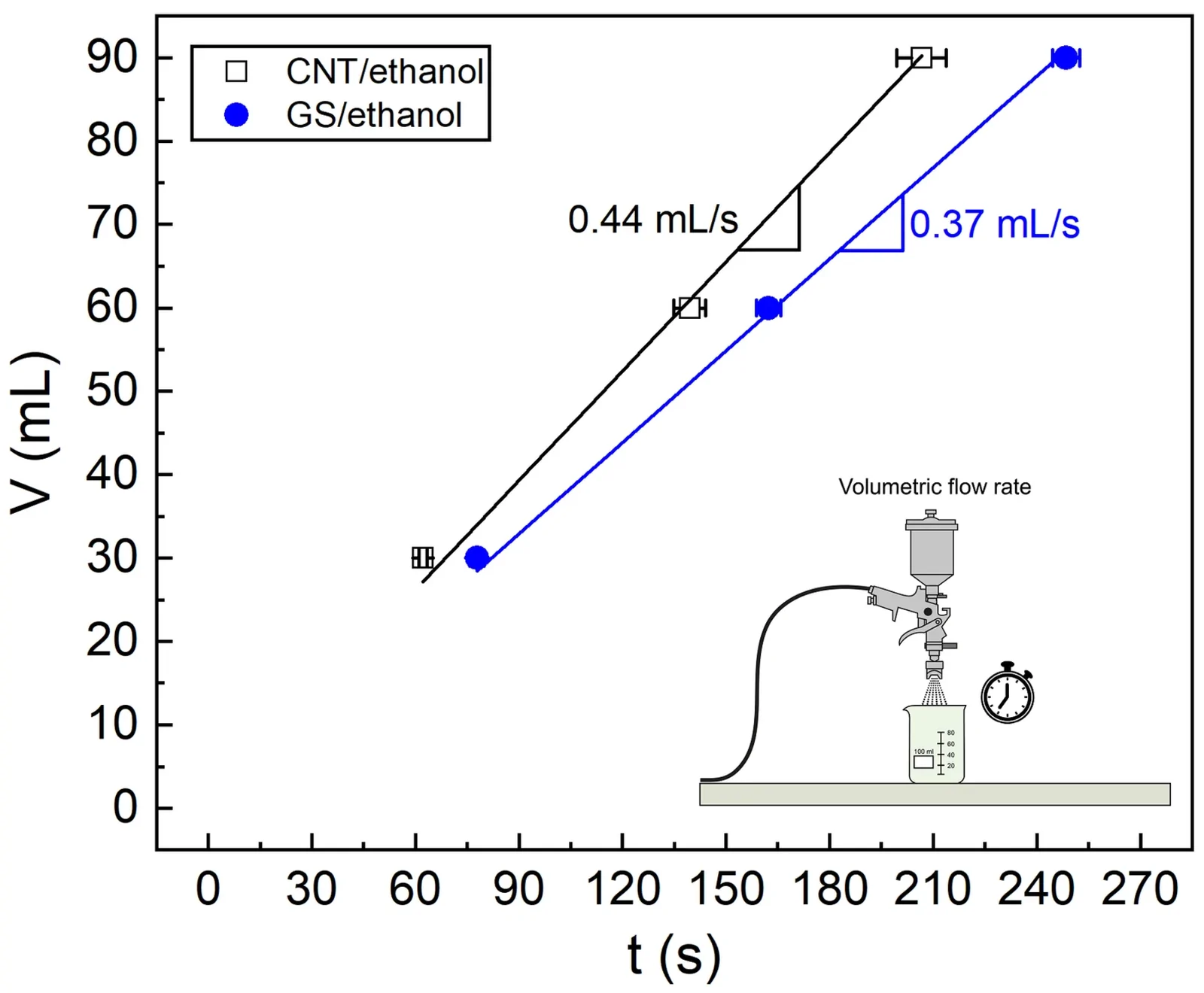
The volume vs. time plot for the three evaluated volumes.

Figure 4 displays the time (*t*) required to deplete the three selected volumes (*V* = 30 mL, 60 mL, 90 mL) of CNT/ethanol and GS/ethanol solutions using the air-spray gun, under identical conditions of the spray gun.

A linear relationship is observed between depleted volume and elapsed time for both solutions. The slope of each line represents the time required for a volumetric unit of the CN solution to flow through the air gun. The volumetric flow rate is 0.44 (±0.01) mL/s for CNTs in ethanol and 0.37 (±0.01) mL/s for GSs in the same ethanol. For the tested volumes, the coefficient of variation of both volumetric flows remained below 2.7% for all cases. This high reproducibility indicates that the flow rate is practically constant for the range of volumes examined, promoting a uniform deposit of CNs on the GFWs.

Since identical set-up conditions of the air gun were used, a comparison of the air flow characteristics of the CNTs and GS can be conducted. The observed differences in the flow rate are mainly attributed to the different morphology of CNs and the higher concentration of GSs (4 mg/mL) compared to that of CNTs (1 mg/mL), which generate different viscous flow conditions. Besides the higher concentration of GSs, their two-dimensional topology and significantly higher aspect ratio (see dimensions in Section 2.1) yield higher excluded volume than CNTs [35,36], increasing nanoparticle entanglement and hence viscosity.

The excluded volume is defined as the volume around a solid object into which the center of another one cannot penetrate. It represents the space between fillers due to their geometry and higher values indicate more loosely packed conductive networks.

Given the geometric dimensions of each CN investigated herein, the excluded volume of CNTs ($V_{Exc}^{CNT}$) and GSs ($V_{Exc}^{GS}$) can be estimated as [36]

(1a) $$V_{Exc}^{CNT}=\left(\frac{\pi }{2}\right)L_{CNT}^{2}d_{CNT}$$

(1b) $$V_{Exc}^{GS}=\left(\frac{\pi^{2}}{8}\right)d_{GS}^{3}$$

Using Equation (1a,b) and the dimensions of the CNs listed in Section 2.1, the estimated excluded volume of GSs is ~10^−14^ m^3^, while that of CNTs is ~10^−19^ m^3^. Thus, $V_{Exc}^{CNT}$/$V_{Exc}^{GS}$ ~ 10^−5^ indicating that the CNTs yield a denser packed network than the GSs.

Previous studies have reported that operating conditions produce different flow rates when coating substrates with CNs using spraying techniques. Depending on the specific coating method, flow rates may range from 10^−5^ mL/s for electrohydrodynamic atomization deposition [37,38] to 10^1^ mL/s for spray pyrolysis [39]. Although the volumetric flow rate is certainly influenced by nozzle aperture and air-gun settings, as well as particle concentration and dispersion state, the volumetric flow rates obtained in this study (10^−1^ mL/s) are within the range of those reported for the spraying methods mentioned above.

A strong dependence of the coating properties on volumetric flow rate has been observed for spray techniques [40], indicating the importance of controlling and quantifying this parameter to achieve homogeneous and property-tailored coatings. For instance, Castillo et al. [38] observed that higher flow rates yield denser and less porous coatings. Therefore, reporting the volumetric flow rate is recommended here as a quantitative metric to support the standardization of coating control protocols and facilitate universal comparison across studies. A linear trend in the volumetric flow rate such as those shown in Figure 4 may be indicative of homogeneous dispersion of the CNs, as reflected in the electrical resistance distribution maps and microscopy images discussed later.

### 3.2. Spot Size

**Figure 5:**
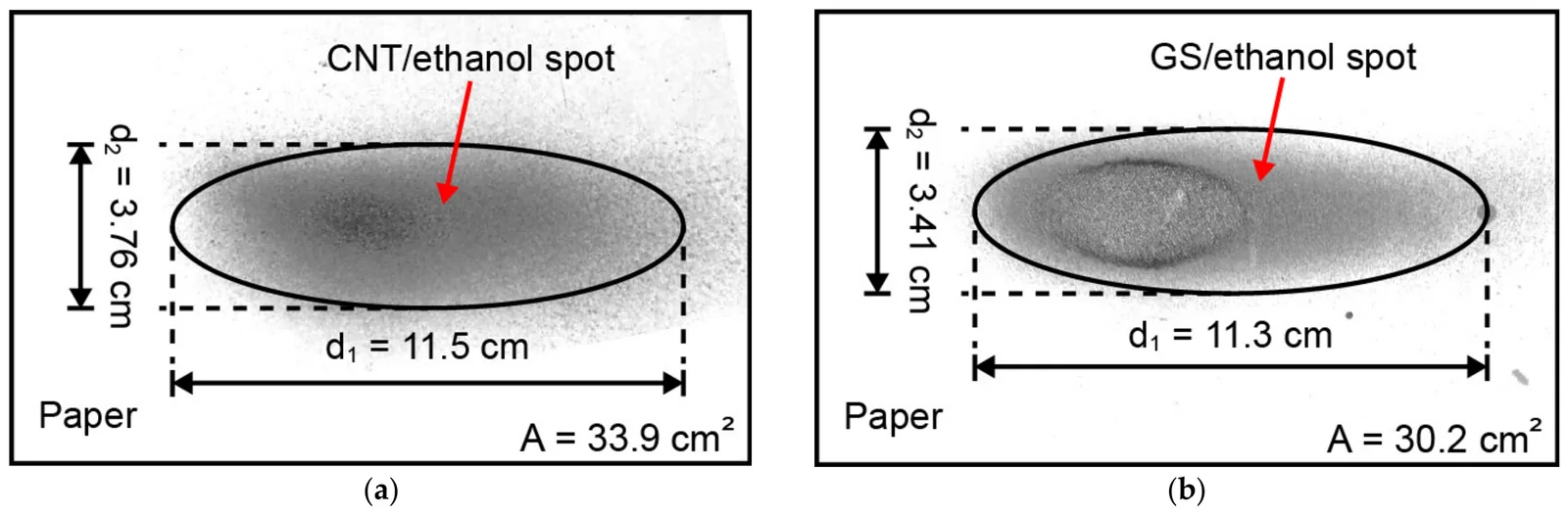
The measurement of the air-gun spot size: (**a**) CNT/ethanol, (**b**) GS/ethanol.

A second parameter that can be used for characterization and quality control assessment of the air-spraying method of carbon nanostructures is the size of the spot left on the substrate. The results of the air-gun spot size estimation of the CN/ethanol solution are displayed in Figure 5a, while those of the GS/ethanol solution are shown in Figure 5b.

The spots produced by both CN/ethanol solutions can be assumed as ellipses. The elliptical shape is due to the flat-fan geometry of the nozzle of the commercial air gun. Measurements performed with the Image J^®^ software show that the mean spot area left on the printing paper was 33.9 (±2.57) cm^2^ for CNTs and 31.3 (±3.57) cm^2^ for GSs, using a three-replicate test program. The mean major axis (*d*_1_) is 11.5 (±0.51) cm for CNTs and 11.6 (±1.37) cm for GSs, while the minor axis (*d*_2_) is 3.76 (±0.13) cm for CNTs and 3.45 (±0.04) cm for GSs. The relatively low coefficients of variation (less than 12%) for the area and major and minor axes of the ellipses indicate a reproducibility of the process. Since the lateral shifts of the serpentine trajectory (0.2 cm) are smaller than the major axis of the spot size (11.5 cm), adjacent passes are expected to overlap and deposit multiple layers of CNs on the same GF surface. This overlap promotes the formation of a continuous, saturated percolation network, yielding a homogeneous distribution of electrical resistance, as will be discussed.

Regarding reported values of spot size, Kim et al. [41] evaluated the variation of this parameter as a function of the distance of the nozzle to the substrate in spray printing with conductive inks. For a nozzle diameter of 0.4 mm with a hollow-cone geometry, the authors observed a spot size of ~34 mm at 10 cm standoff distance, with the spot diameter increasing at larger distances. Furthermore, the hollow-cone spray produced a ring-graded spot characterized by a lower ink concentration and intensity at the center. While hollow-cone nozzles tend to produce a compact, radially symmetric spot, larger flat-fan geometries yield expansive and elongated spots [41], highlighting how different nozzle geometries (flat-fan in this work) dictate the spot layout and overall uniformity of the conductive network.

### 3.3. Areal Density

**Figure 6:**
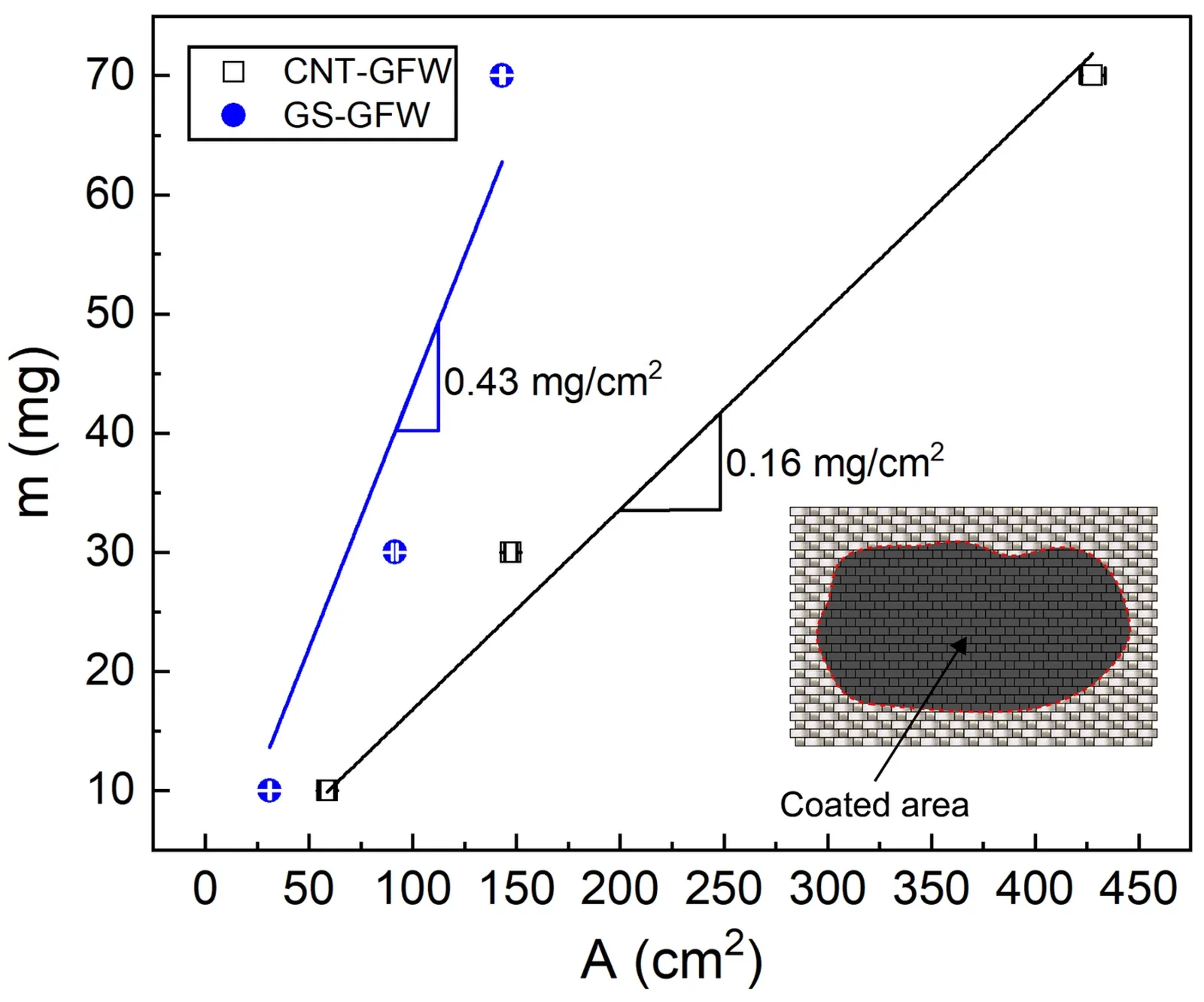
Mass vs. area for the determination of the CN areal density.

The substrate area covered by the CNs for a given amount (mass) of CNs is a key parameter for characterizing surface coating, which has also been scarcely reported in the literature. Figure 6 presents the GFW surface covered area for three masses of CNTs and GSs, viz., 10 mg, 30 mg and 70 mg. The areal density for each CN was determined as the slope of the linear fit of the covered area (*A*) vs. mass plot, as depicted in Figure 6.

The areal density was 0.16 (±0.01) mg/cm^2^ for the CNTs and 0.43 (±0.05) mg/cm^2^ for the GSs. These values should be taken as upper bounds since small mass losses, such as CN material stuck in the air-gun container and conduits after fluid depletion, are not considered. Those mass losses are expected to increase with increased initial CN content, which may cause the deviation from linearity observed in Figure 6. The observed difference between CNTs and GSs is partly attributed to the higher weight concentration in solution of GSs with respect to CNTs (4:1). However, the areal density of the GS coating is 2.7 times higher than that of the CNT coating, which does not match the 4:1 mass ratio of the actual mass of CNs used for each coating. These results imply a lower efficiency of the GS deposition to cover a given area compared to CNTs. This reduced efficiency is again explained by the significantly higher excluded volume of the GSs when compared- to that of the CNTs (see calculations in Section 3.1), resulting in a less efficient packing density [35,36].

Shou et al. [42] systematically evaluated the effects of the air-gun speed, air pressure, and the number of sprayed layers on the areal density of carbon nanofiber-based coatings. By studying those parameters, the quantified areal densities were between 0.01 and 0.27 mg/cm^2^, which is lower than the areal density determined in this work for GSs (0.43 mg/cm^2^) and similar to that for CNTs (0.16 mg/cm^2^). The authors observed that the areal density decreases with the speed of the air gun during spraying and the air pressure while increasing with the number of sprayed layers. In another work [38], the volumetric density of a carbon-black coating was estimated by measuring the thickness of the coating, observing an increase of the volumetric density with higher volumetric flow rates. Although this metric may be comparable to areal density, measuring thickness can be more complex and less practical.

### 3.4. Morphology and Thickness of Coated Glass Fiber

**Figure 7:**
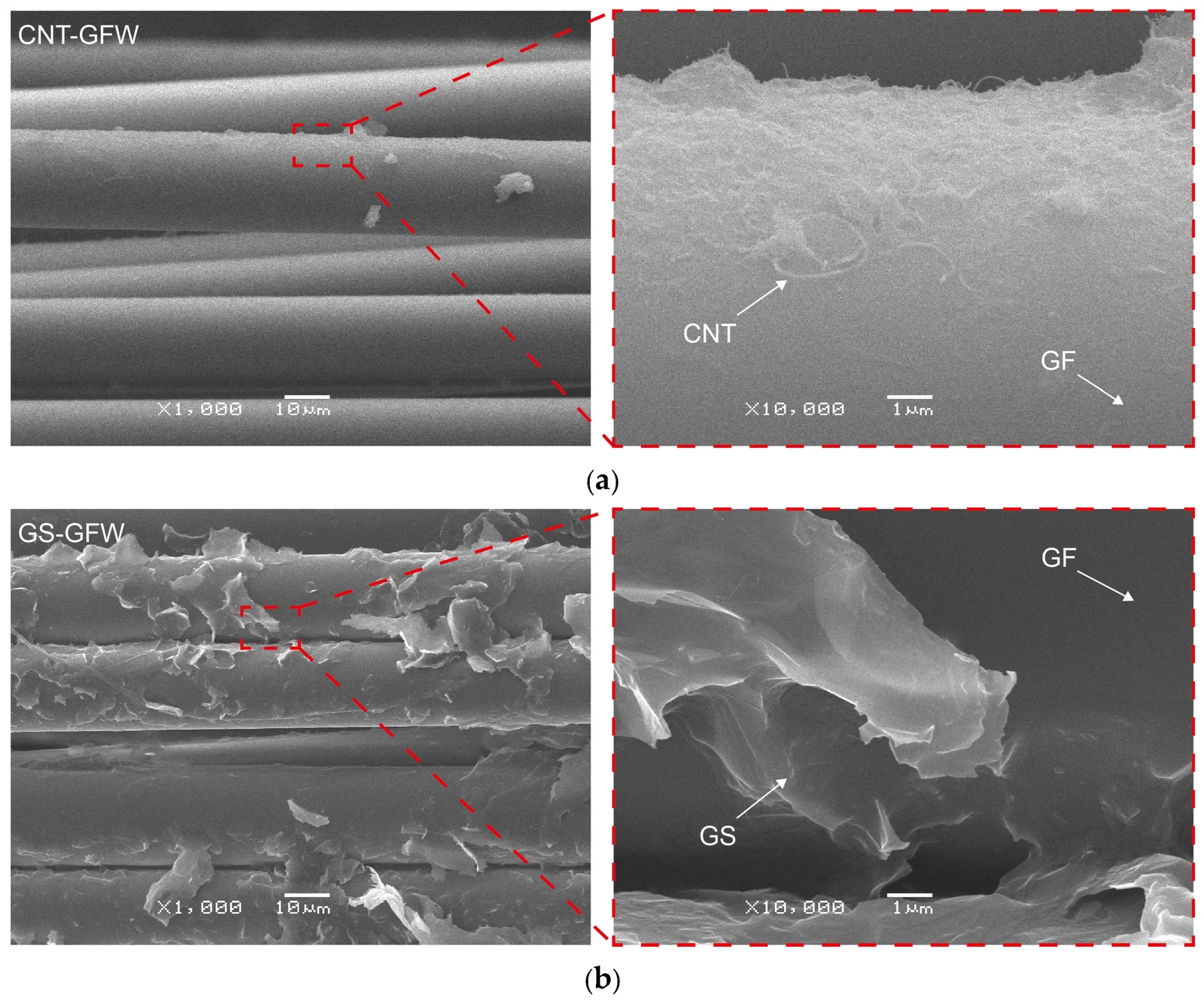
SEM images of CN-coated GFWs: (**a**) CNTs, (**b**) GSs.

SEM and AFM analyses were conducted to observe the distribution of the CNs onto the GFW surface. Figure 7 displays SEM images of both CNT and GS distributions on the GFWs at two magnifications.

A uniform distribution of CNTs coating the GFW surface is observed in the SEM micrographs at 1000× and 10,000× magnifications (Figure 7a), with the GF surface fully covered with randomly oriented CNTs. In marked contrast, the GS coating (Figure 7b) is more irregular and inhomogeneous, with localized agglomerated regions. This is mainly because of the two-dimensional geometry and higher excluded volume of the GSs, which render less efficient packing densities [35,36]. Processing the GSs at the same concentration and energy density as CNTs yielded significantly more agglomerated nanostructures and heterogeneously covered fibers, not suitable for this application. It is also important to keep in mind that both CNs were used as-received, and their amount of functional groups that can react with the glass fiber surface is limited [32,43,44,45]. It has been proven that chemical functionalization of GSs can greatly enhance their dispersion and physicochemical interaction with inorganic substrates [46,47], but this strategy falls out of the scope of this work.

**Figure 8:**
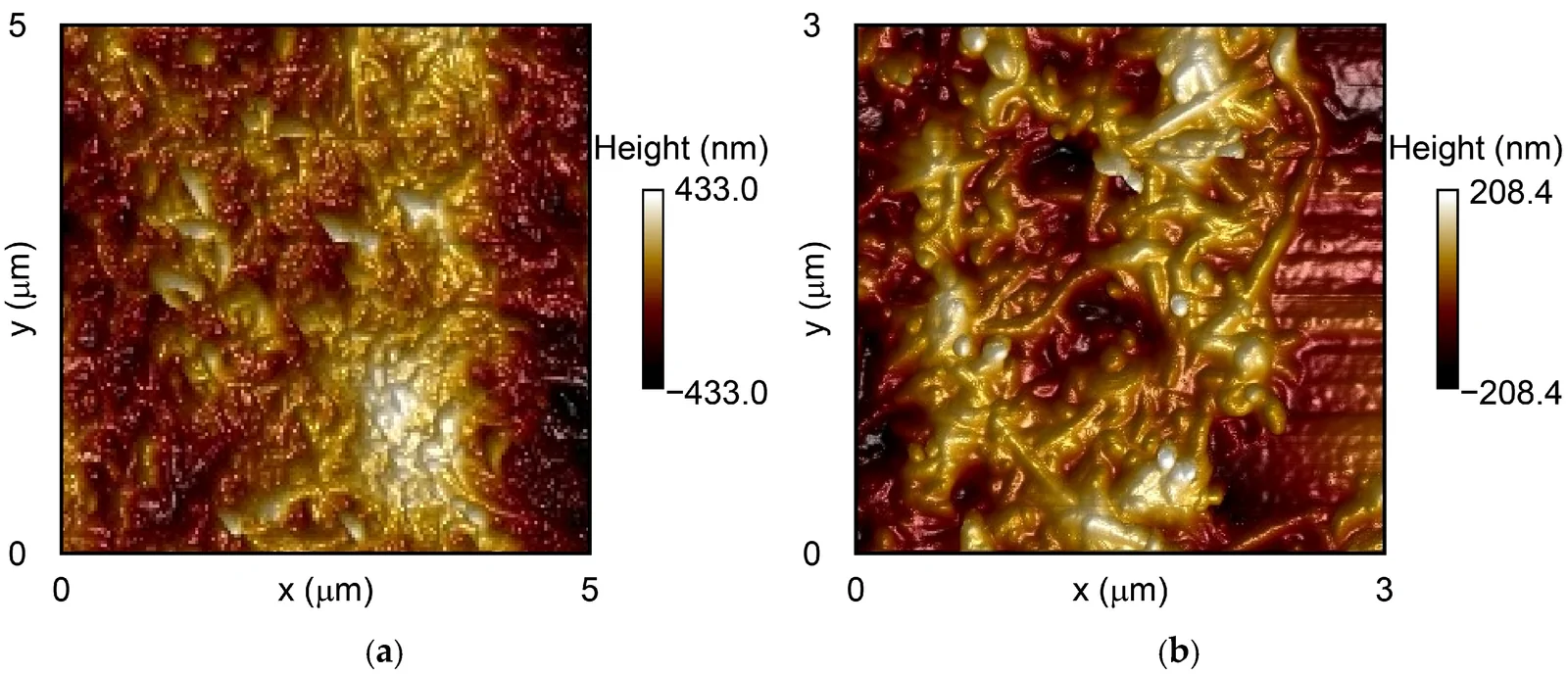
AFM topographic images of CNT-coated GFWs: (**a**) 5 µm size, (**b**) 3 µm size.

The surface topography of the CNT-coated GFWs is illustrated in the AFM micrographs of Figure 8. Figure 8a shows the topography of CNT coating across a 5 µm × 5 µm scan area, while Figure 8b provides a higher-resolution view (3 µm × 3 µm), highlighting the detailed entanglement of the CNTs.

Figure 8 reveals that CNTs uniformly coat the surface of the GFWs, even at the microscale. The measured RMS surface roughness was 17.2 ± 6.2 nm. The surface roughness is comparable to that reported in previous studies. For instance, Ku-Herrera et al. [31] coated GFWs with CNTs using the dipping method and various solvents for CNT suspension. When water was used as the dispersion medium, the average RMS roughness was 26.7 nm. In contrast, when hydrolyzed silane was employed as suspending medium, the average RMS roughness was approximately 17.8 nm. In a related study, Zhang et al. [6] deposited carboxyl-functionalized CNTs onto GFs using both electrophoretic and dip coating methods. AFM analysis showed that the electrophoretic method yielded an average roughness of 20.6 nm, compared to 15.3 nm for the dip-coated specimens. However, the electrophoretic method produced a more homogeneous CNT network onto the GFW surface than the dipping method, with less uncovered surface.

The comparatively lower RMS surface roughness (17.2 nm) achieved in this work is related to the homogeneous dispersion and distribution of CNTs, which minimizes the agglomeration of these CNs and produces a smoother surface. Particle concentration in solution and the energy density used for dispersion may also influence surface roughness.

AFM imaging of the GS coatings was not possible because it exceeded the dynamic range of the scanner. The very large surface height variations and roughness of the GS coatings often exceeded the 2.5 µm *z*-axis limit of the scanner, resulting in piezo-saturation and preventing the acquisition of reliable images.

**Figure 9:**
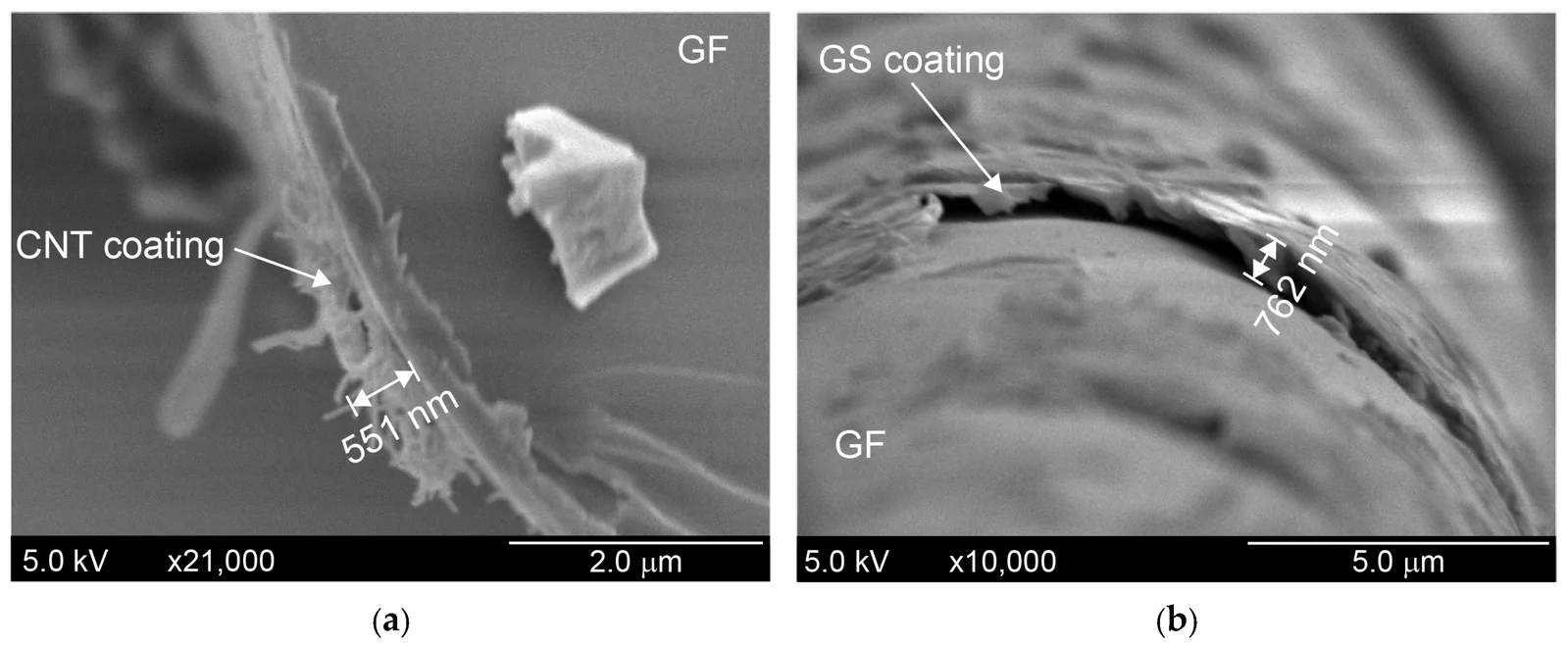
High-resolution SEM micrographs used for measuring coating thickness: (**a**) CNT coating. (**b**) GS coating.

Representative high-resolution SEM micrographs used for the estimation of coatings thickness are depicted in Figure 9.

Measurements of several coated fibers indicate that the thickness of the CNT coating (Figure 9a) ranges between 500 and 650 nm. Consistent with the previous SEM and AFM findings, the thickness of the GS coating (Figure 9b) is higher and has a wider distribution range, ranging between 500 and 800 nm. As discussed previously (see Section 3.1), the higher GS thickness can be attributed to the higher excluded volume generated by these nanostructures [36] and also probably due to the higher concentration of GSs used. This thickness correlates with the areal density determined for each coating in Figure 6. For the same surface area of GFW, the GS coating has more nanostructures on the fibers than the CNT one (due to their higher concentration), leading to a thicker coating.

### 3.5. Raman Spectra of Coated Glass Fibers

**Figure 10:**
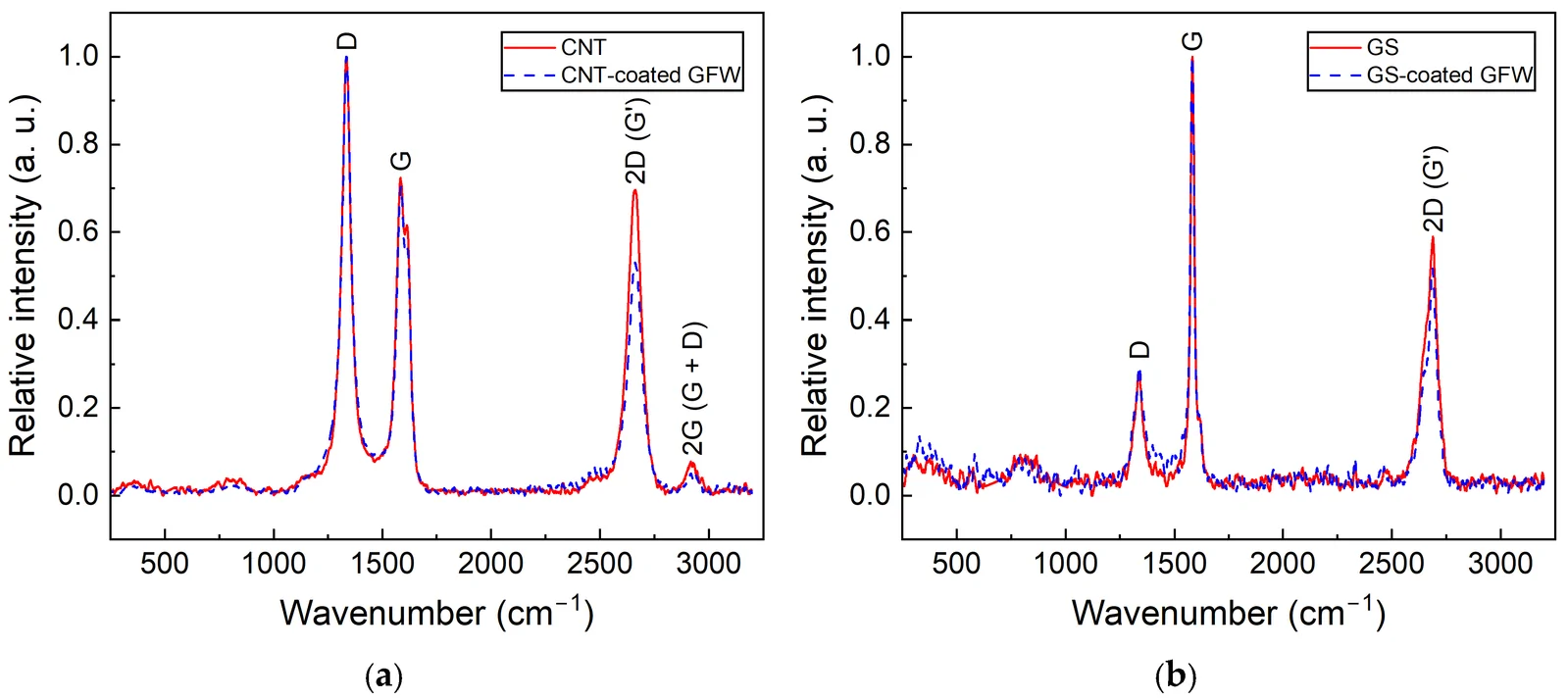
Raman spectra of CNs before and after coating the GFW surface: (**a**) Neat CNT and CNT-coated GFW, (**b**) Neat GS and GS-coated GFW.

Figure 10 presents the Raman spectra of CNTs (Figure 10a) and GSs (Figure 10b) comparing the spectra before and after ultrasonic dispersion. The reference spectrum (labeled either CNT or GS) corresponds to the neat, as-received nanostructure. The second spectrum (in dashed blue line) corresponds to the CNs covering the GFW, i.e., after they have been dispersed by ultrasonic energy and coated the fiber surface.

Three main bands are observed in Figure 10 for both CNs at 1350 cm^−1^ (*D*), 1580 cm^−1^ (*G*) and 2700 cm^−1^ (2*D*) [32,48]. For CNTs, an additional band of significantly smaller intensity is observed at 2935 cm^−1^, corresponding to the *G* + *D* band [48]. Band intensities were normalized to the *D* band and fitted to Gaussian functions to determine their relative intensities. The intensity ratio between *D* and *G* bands (*I_D_*/*I_G_*) was calculated from six specimens of each CN type, both as-received and after ultrasonic dispersion and deposition onto fibers. The *I_D_*/*I_G_* ratio for as-received CNTs was 1.37 ± 0.06 (mean ± standard deviation), while the one measured on the CNT-coated fiber was 1.38 ± 0.09. This indicates that CNTs maintain their structural integrity after the process of GFW coating. For GSs, the *I_D_*/*I_G_* was 0.25 ± 0.03 for as-received GSs and 0.30 ± 0.09 when measured over the coated fibers (i.e., after ultrasonic dispersion and spraying). This small increment in the average value suggests that a slight increase in edge defects within the graphenic structure might be possible. This may be explained by the higher energy density employed for dispersion of GSs compared to CNTs. However, considering the standard deviation overlapping, the difference is not significant. The absence of important changes between the Raman spectra before and after the coating process indicates that the ultrasonic dispersion and air-spraying parameters used herein do not degrade the structural integrity of the CNs, preserving their intrinsic and structural properties upon transferring to the GFW. This is another metric that can be used for quality control and assessment of the air-spraying method, as a non-destructive tool for fiber coating.

### 3.6. Adhesion of Coatings to Glass Fiber Weaves

**Figure 11:**
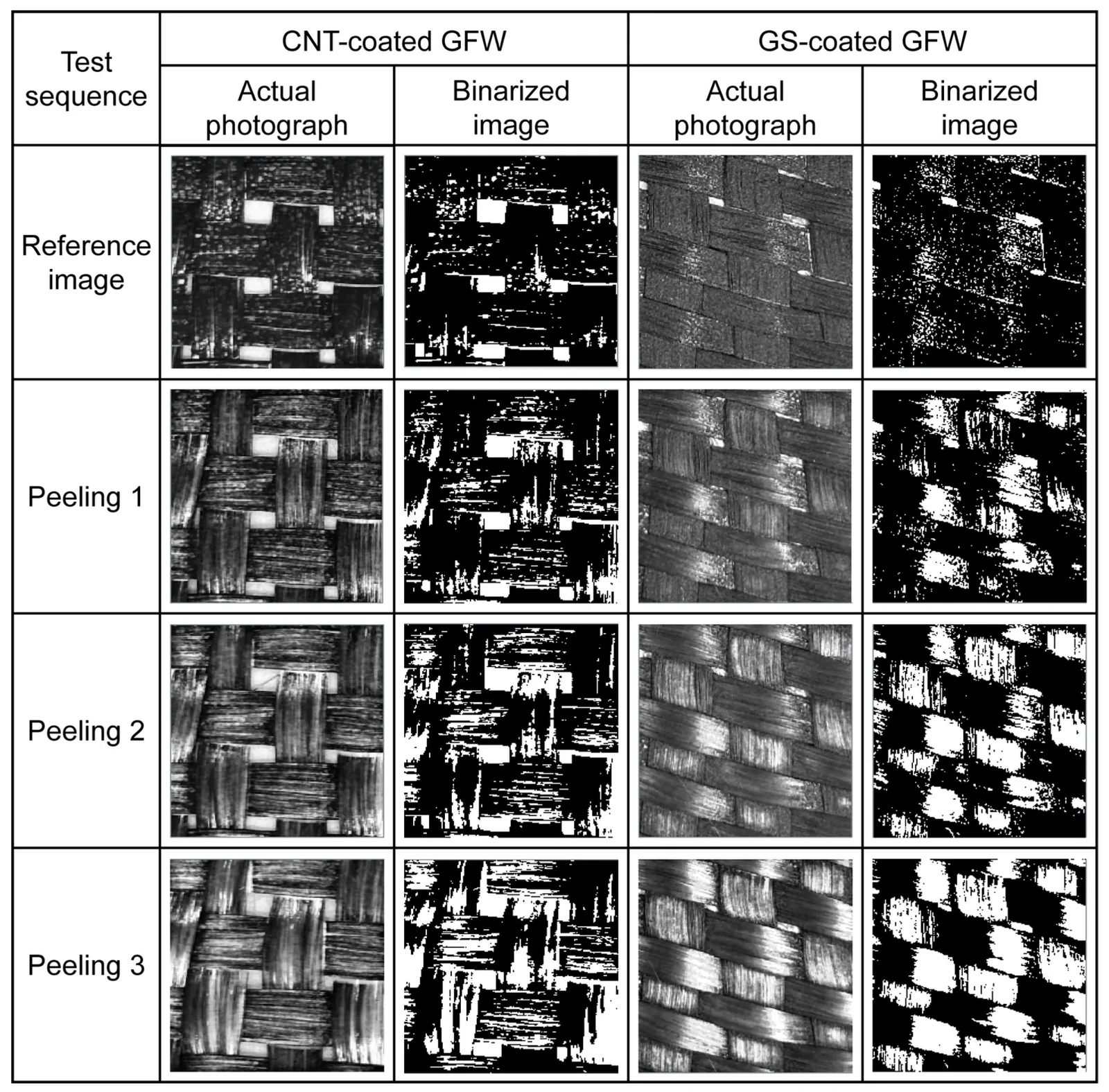
Photographs and binarized images of CNT-coated GFW and GS-coated GFW specimens before and after the peeling tests.

Figure 11 presents representative optical images (as-taken and binarized) used to assess the adhesion of CNT and GS coatings to the GFWs.

**Figure 12:**
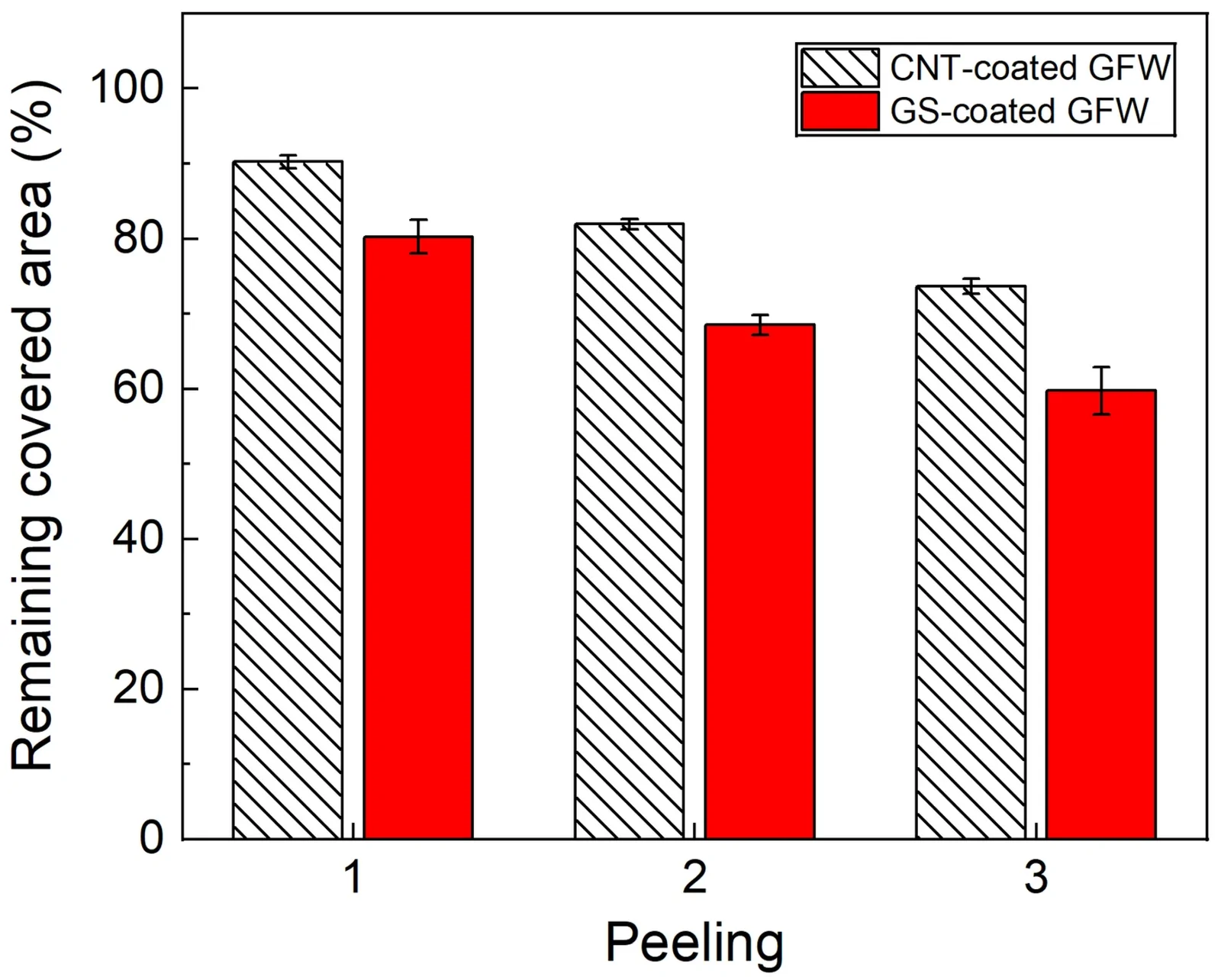
Remaining CN-covered area after each sequential peeling test on coated GFWs.

The reference images display a dark, uniform black color. The loss of material, i.e., the detachment of CNs, is more evident for the GS-coated fibers and after the third sequential peeling. Surface adhesion of the CNs was quantified using a dedicated pixel-counting software, which compared the area covered with CNs of the reference image (before peeling) with the area covered after each peeling test. The results of this relative quantification are presented in Figure 12.

Consistent with the visual inspection, the quantification indicates that after the first peeling, the remnant area coated with CNTs was 90.2 (±0.9) % and 80.3 (±2.3) % for GSs. After subsequent peelings, the remnant covered area decreased up to 73.7 (±1.0) % for CNTs and 59.8 (±3.2) % for GSs. In general, after the third peeling, all coated GFWs maintain a covered area above 50%, indicating a relatively good adhesion of the CNs to the GFW surface. For the three sequential peelings, adhesion was always higher for CNT-coated fibers.

There are two major factors that contribute to CN-to-glass fiber adhesion, viz., a chemical component and mechanical interlocking. From the former perspective, previous studies by our group [19] have demonstrated that the organic coating (sizing) of the GFWs used in this work comprises oxygen-containing functional groups (carboxylic, hydroxyl, and epoxy) that interact with the functional groups present on the surface of the CNs [31]. The commercial CNs used herein have also been previously characterized by our group. In the as-received CNTs, moderate levels of -OH (hydroxyl) and -COOH (carboxylic) functional groups are introduced during their synthesis and purification [19]. Similarly, previous physicochemical characterization of the GSs indicates the presence of hydroxyl and carboxylic acid functionalities [47]. The average *I_D_*/*I_G_* ratio of the as-received CNTs is 1.37, while that of the GSs used here is only 0.25. The Brunauer, Emmett, and Teller (BET) specific surface area of the CNTs used here is 110 m^2^/g [43,44], while that of the GSs (H25) is only ~49 m^2^/g [32,45]. X-ray photoelectron spectroscopy and Fourier transform infrared spectroscopy of the CNs used herein have shown that both nanostructures present oxygen-containing functionalities in their surface, such as hydroxyl, methylene, carbonyl, and epoxides [32,44]. Our previous studies show that the density of oxygen-containing functionalities, given by their C/O ratio, is also significantly lower for the GSs used in this research (34.4) than for the CNTs (12.3) [32,44]. Therefore, for the specific CNTs and GSs used herein, chemical affinity with the GF surface is expected to be higher for CNTs. From the perspective of mechanical interlocking, the significantly higher excluded volume of GSs with respect to CNTs (10^5^ times higher) yields a more loosely packed coating for GSs, which can be more easily removed during the peeling tests. The higher (BET) surface area of the CNTs might also render greater interfacial contact area and hence stronger mechanical interlocking.

### 3.7. Electrical Resistance Distribution of Coated Glass Fiber Weaves

**Figure 13:**
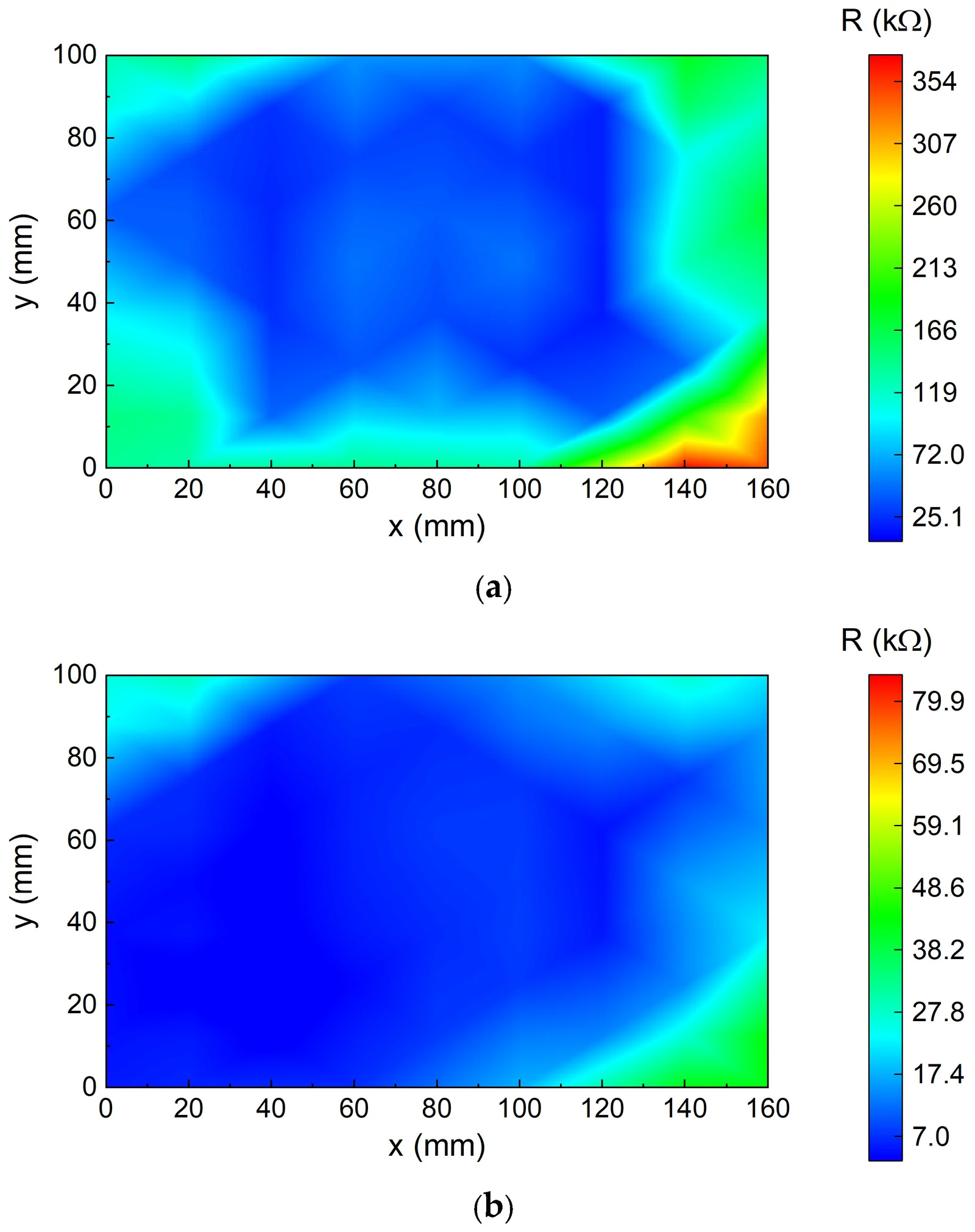
Electrical resistance distribution isocontour maps: (**a**) CNT-coated GFW. (**b**) GS-coated GFW.

Figure 13 exhibits representative isocontour maps of the electrical resistance distribution for a CNT-coated GFW (Figure 13a) and for a GS-coated GFW (Figure 13b).

For GFWs coated with CNTs, the isocontour map displays electrical resistances ranging from 25 to 354 kΩ. The central region of the modified GFWs exhibits a uniform *R* distribution below 98.9 kΩ (blue). Higher *R* values (up to 354 kΩ) are only observed at the edges of the specimen, especially near the corners at *x* = 160 mm and *y* = 0 mm.

For GFWs coated with GSs, the electrical resistance distribution ranges from 3.7 to 41.1 kΩ. Similar to the CNT-coated GFW, the central region shows a uniform distribution of *R* with values below 17.4 kΩ, while the edges exhibit higher gradients with values up to 59.1 kΩ, specifically at one corner of the specimen (*x* = 160 mm, *y* = 160 mm). This indicates that both CN concentrations are above the percolation threshold, resulting in a conductive network with redundant paths. GS-coated GFWs presented a range with slightly lower electrical resistances. The resistance values normalized by the distance between electrodes was determined for each coating, finding that the CNT coatings have resistance between 6.3 × 10^2^ kΩ/m and 1.3 × 10^4^ kΩ/m. The normalized resistance of the GS coatings is lower, ranging from 53 kΩ/m to 1.1 × 10^3^ kΩ/m, which is attributed to the higher concentration of these nanostructures (4:1 by weight). The fact that a concentration of GSs, which is four times higher than that of CNTs (see Section 2.2), is needed to achieve similar or slightly lower electrical resistances means that the CNTs used here produce more efficient electroconductive networks than GSs relative to the amount of deposited material. This is likely a geometric effect that could be explained by the excluded volume theory. Due to their geometry, the excluded volume of GSs is five orders of magnitude higher than that of CNTs (see calculations in Section 3.1), indicating that CNTs generate a more densely packed network at the same weight concentration of nanostructures. This is clearly seen in the micrographs of Figure 7. Furthermore, the higher BET surface area of CNTs may potentially provide more contact opportunities between nanostructures, improving the electrical transport efficiency within the network.

### 3.8. Application to Electrical Damage Detection in Laminated Composites

**Figure 14:**
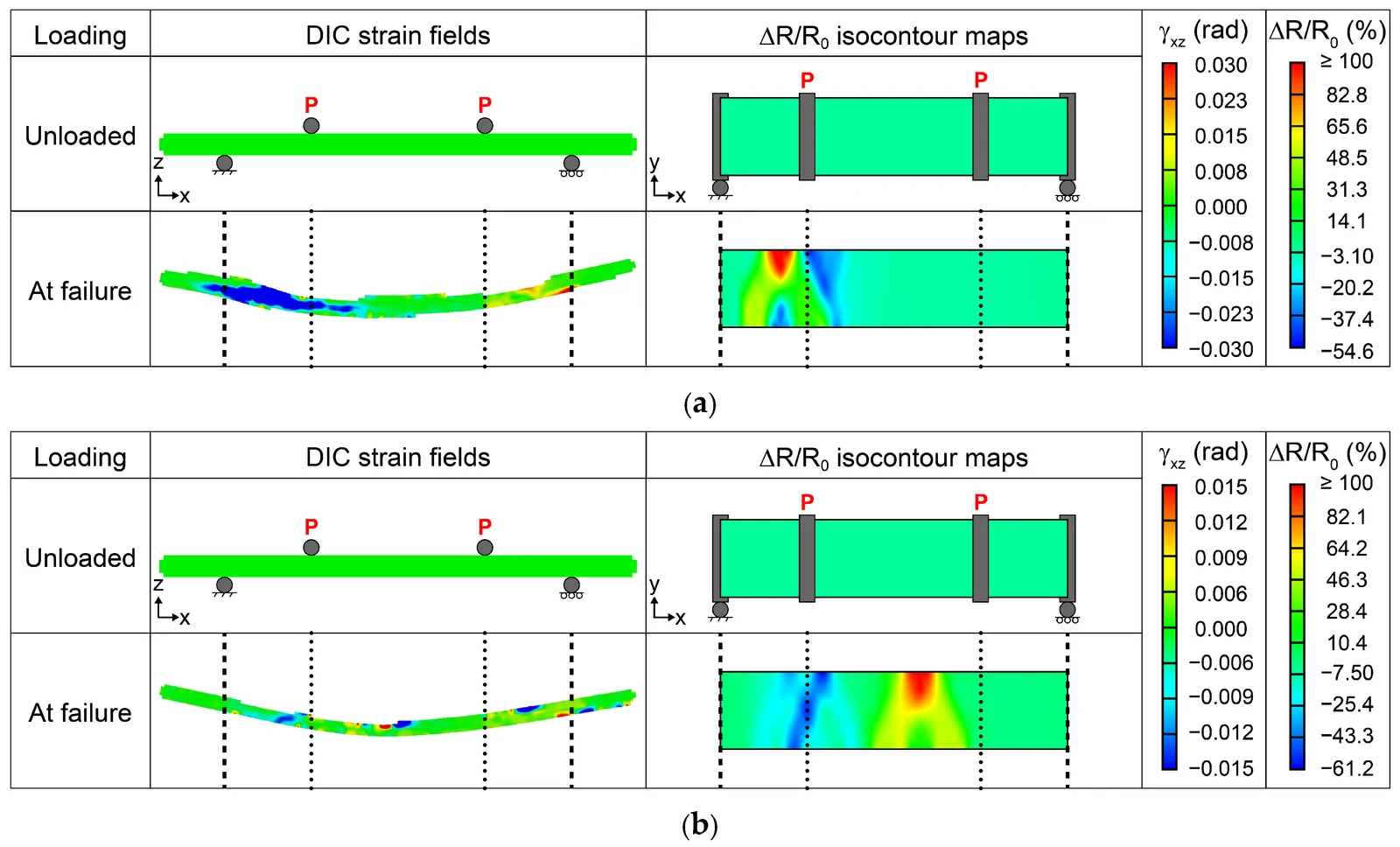
Electrical monitoring of damage of two representative hierarchical composite specimens at failure. The second column represents the DIC strain fields, while the third column the Δ*R*/*R*_0_ isocontour maps: (**a**) CNT-coated specimen, (**b**) GS-coated specimen.

Figure 14 summarizes the results of electrical monitoring of damage in laminated composite beams using one layer of GFWs coated with CNTs (Figure 14a) and GSs (Figure 14b) for a monotonic bending test. The isocontour plots of fractional change of electrical resistance (Δ*R*/*R*_0_) correspond to the unloaded state and at the failure of the composite (when the maximum stress was applied).

At failure, the laminated composite containing a CNT-coated GFW (Figure 14a) shows a positive maximum Δ*R*/*R*_0_ above 100% under the left load introduction element, which extended close to the left support. A region with maximum negative Δ*R*/*R*_0_ ~ −54.6% is seen at the *y*-opposite edge, which can be associated with compression failure under the left load introduction element. DIC fields also indicate that the highest transverse shear strains (*γ_xz_*) occur in the region between the left load introduction element and the support.

Since failure in composites is stochastic, a different specimen containing one layer of GS-coated GFW (Figure 14b) shows critical failure zones at different locations, as evidenced by the DIC patterns of *γ_xz_* in the last row of Figure 14. The locations with the highest *γ_xz_* also show a strong correlation with those of highest Δ*R*/*R*_0_. The central region presents a maximum Δ*R*/*R*_0_ above 100%, while the highest negative values (−61.2%) are located under the left load introduction element. These findings show that both coatings exhibit high sensitivity to damage detection, with similar electrical resistance changes. However, the content of the CNTs is 4 times lower (in weight) than the content of GSs, indicating the formation of a more efficient network for the former. The GS-modified composites presented slightly higher negative Δ*R*/*R*_0_, which can be attributed to the platelet morphology of the GSs, leading to a loosely packed conductive network. This observation aligns with the microscopic examination of the coatings in Figure 7 and with the excluded volume theory explained earlier, where the CNTs form a uniform and well-packed network, whereas GS coatings exhibited a loosely packed one, less homogeneous and with larger agglomerates. Similar observations were found in [13], where GS-coated fibers presented higher sensitivity to damage caused by monotonic tension. In that study, higher sensitivity was attributed to the laminated structure of the sensing interphase created by GSs on the fiber surface compared with the tangled coating of CNTs. Although GS coatings may require a higher amount of nanomaterial than CNTs to achieve comparable coating’s electrical conductivity, their stacked network architecture may offer opportunities to tailor the electromechanical response of self-sensing composites. Less densely packed GS networks may exhibit enhanced electrical sensitivity to damage. In addition, the higher excluded volume of GS coatings might promote greater interaction with the polymer matrix, potentially enhancing their ability to better detect interfacial and matrix-dominated failure mechanisms in polymer composites.

## 4. Conclusions

Glass fiber weaves (GFWs) were coated with either carbon nanotubes (CNTs) or multilayer graphene sheets (GSs) to render electrically conductive materials. The coating conditions were analyzed to determine the volumetric flow rate, areal density, and the spot size of the air gun used for the coatings. The coatings were also characterized to determine the coating thickness, retention of structural integrity of the nanostructures upon coating, adhesion of the carbon nanostructures to the glass fibers, and electrical resistance spatial distribution over the GFWs. Since the key practical use here focuses on electrical conductivity, the concentrations of CNTs and GSs in the spraying solution were adjusted to achieve similar values of electrical resistance in the coated GFWs. This required four times less weight material for CNTs than for GSs and also significantly higher dispersion energy density for GSs. Overall, CNTs produced a more uniform coating, which was more densely packed and had better adhesion to the GFWs. The CNT coatings were also thinner (~500–650 nm) than the GS ones and had significantly less surface roughness (17.2 ± 6.2 nm). These results are deemed to be strongly influenced by the excluded volume generated by both carbon nanostructures upon coating the fiber surface, which, given their dimensions, is around five orders of magnitude greater for GSs than for the CNTs used herein. Better adhesion to GFWs was obtained for CNT coatings than for GS ones. This is presumably attributed to stronger physicochemical interactions with the GFW sizing due to their higher density of oxygen-containing functional groups, probably complemented with a geometric interlocking effect. While both carbon nanostructures enabled effective damage detection when incorporated into smart laminated polymer composites, GS-modified laminates exhibited slightly higher electrical sensitivity due to their looser packing. However, CNT-modified specimens required four times less nanostructure content (by weight) to achieve comparable sensitivity.

The current work proposes a set of quantitative metrics and tools for quality-control assessment of the air-spraying process used to coat engineering fibers with carbon nanostructures, which could lay the groundwork for future standardization. The findings point out that the quality and electrical conductivity of the surface coating depend strongly on the morphology, concentration, dispersion state, and physicochemical properties of the nanostructures used.

## Data Availability

The original contributions presented in this study are included in the article material. Further inquiries can be directed to the corresponding author.

## References

[1] Moriche R., Jiménez-Suárez A., Sánchez M., Prolongo S.G., Ureña A. (2018). Sensitivity, Influence of the Strain Rate and Reversibility of GNPs Based Multiscale Composite Materials for High Sensitive Strain Sensors. Compos. Sci. Technol..

[2] Al-Bahrani M., Cree A. (2019). A Simple Criterion to Evaluate the Degree of Damage in Composite Materials after Sudden Impact Loads by Exploiting the MWCNTs Piezoresistive Property. Carbon.

[3] O’Donnell J., Chalivendra V., Hall A., Kim Y. (2020). Damage Sensing in Multi-Functional Glass Fiber Composites under Mode-I Fracture Loading. J. Compos. Mater..

[4] Lopes C., Araújo A., Silva F., Pappas P.N., Termine S., Trompeta A.F.A., Charitidis C.A., Martins C., Mould S.T., Santos R.M. (2024). Smart Carbon Fiber-Reinforced Polymer Composites for Damage Sensing and on-Line Structural Health Monitoring Applications. Polymers.

[5] Gu W., Jin X., Zhang J., Guo N., Shi Y., Shi J., Lan X. (2026). Carbon-Material-Modified Polyester Nonwoven Composites with Enhanced Mechanical, Electrical, and Thermal Properties. Polymers.

[6] Zhang J., Zhuang R., Liu J., Mäder E., Heinrich G., Gao S.L. (2010). Functional Interphases with Multi-Walled Carbon Nanotubes in Glass Fibre/Epoxy Composites. Carbon.

[7] An Q., Rider A.N., Thostenson E.T. (2013). Hierarchical Composite Structures Prepared by Electrophoretic Deposition of Carbon Nanotubes onto Glass Fibers. ACS Appl. Mater. Interfaces.

[8] Uribe-Riestra G.C., Ayuso-Faber P., Rivero-Ayala M., Cauich-Cupul J., Gamboa F., Avilés F. (2024). Structural Health Monitoring of Carbon Nanotube-Modified Glass Fiber-Reinforced Polymer Composites by Electrical Resistance Measurements and Digital Image Correlation. Struct. Health Monit..

[9] Zhao Z., Li M., Liu Y., Wang A., Zhou B., Hu J. (2024). Structural Health Monitoring of Glass Fiber-Reinforced Polymer Laminates with Carbon Nanotube-Coated Glass Fiber Sensing Layer after Low-Velocity Impact Using Electrical Resistance Tomography. Nanomaterials.

[10] Wang Y., Hui Y., Chen X., Wen K., Cheng S., Song Q., Gao Y., Zhang J., Shao J. (2024). Multifunctional MXene/Carbon Nanotubes Coated Glass Fiber Sensors for in-Situ Monitoring of Curing Process and Structural Health of Polymeric Composites. Chem. Eng. J..

[11] Luo S., Liu T. (2014). Graphite Nanoplatelet Enabled Embeddable Fiber Sensor for in Situ Curing Monitoring and Structural Health Monitoring of Polymeric Composites. ACS Appl. Mater. Interfaces.

[12] Sebastian J., Schehl N., Bouchard M., Boehle M., Li L., Lagounov A., Lafdi K. (2014). Health Monitoring of Structural Composites with Embedded Carbon Nanotube Coated Glass Fiber Sensors. Carbon.

[13] Hao B., Ma Q., Yang S., Mäder E., Ma P.C. (2016). Comparative Study on Monitoring Structural Damage in Fiber-Reinforced Polymers Using Glass Fibers with Carbon Nanotubes and Graphene Coating. Compos. Sci. Technol..

[14] He D., Fan B., Zhao H., Lu X., Yang M., Liu Y., Bai J. (2017). Design of Electrically Conductive Structural Composites by Modulating Aligned CVD-Grown Carbon Nanotube Length on Glass Fibers. ACS Appl. Mater. Interfaces.

[15] Balaji R., Sasikumar M. (2017). Graphene Based Strain and Damage Prediction System for Polymer Composites. Compos. Part A Appl. Sci. Manuf..

[16] Mahmood H., Vanzetti L., Bersani M., Pegoretti A. (2018). Mechanical Properties and Strain Monitoring of Glass-Epoxy Composites with Graphene-Coated Fibers. Compos. Part A Appl. Sci. Manuf..

[17] Wang G., Wang Y., Zhang P., Zhai Y., Luo Y., Li L., Luo S. (2018). Structure Dependent Properties of Carbon Nanomaterials Enabled Fiber Sensors for in Situ Monitoring of Composites. Compos. Struct..

[18] Müller M.T., Pötzsch H.F., Gohs U., Heinrich G. (2018). Online Structural-Health Monitoring of Glass Fiber-Reinforced Thermoplastics Using Different Carbon Allotropes in the Interphase. Materials.

[19] Ku-Herrera J.J., Avilés F., Nistal A., Cauich-Rodríguez J.V., Rubio F., Rubio J., Bartolo-Pérez P. (2015). Interactions between the Glass Fiber Coating and Oxidized Carbon Nanotubes. Appl. Surf. Sci..

[20] Rodríguez-González J.A., Rubio-González C., Soto-Cajiga J.A. (2019). Piezoresistive Response of Spray-Coated Multiwalled Carbon Nanotube/Glass Fiber/Epoxy Composites under Flexural Loading. Fibers Polym..

[21] Zhang H., Liu Y., Kuwata M., Bilotti E., Peijs T. (2015). Improved Fracture Toughness and Integrated Damage Sensing Capability by Spray Coated CNTs on Carbon Fibre Prepreg. Compos. Part A Appl. Sci. Manuf..

[22] Atiq Ur Rehman M., Chen Q., Braem A., Shaffer M.S.P., Boccaccini A.R. (2021). Electrophoretic Deposition of Carbon Nanotubes: Recent Progress and Remaining Challenges. Int. Mater. Rev..

[23] Rafiee R. (2017). Carbon Nanotube-Reinforced Polymers: From Nanoscale to Macroscale.

[24] Zhu J., Imam A., Crane R., Lozano K., Khabashesku V.N., Barrera E.V. (2007). Processing a Glass Fiber Reinforced Vinyl Ester Composite with Nanotube Enhancement of Interlaminar Shear Strength. Compos. Sci. Technol..

[25] Zhang H., Kuwata M., Bilotti E., Peijs T. (2015). Integrated Damage Sensing in Fibre-Reinforced Composites with Extremely Low Carbon Nanotube Loadings. J. Nanomater..

[26] Jung Y.T., Roh H.D., Lee I.Y., Park Y.B. (2020). Strain Sensing and Progressive Failure Monitoring of Glass-Fiber-Reinforced Composites Using Percolated Carbon Nanotube Networks. Funct. Compos. Struct..

[27] Rubio-González C., de Urquijo-Ventura M.P., Rodríguez-González J.A. (2023). Damage Progression Monitoring Using Self-Sensing Capability and Acoustic Emission on Glass Fiber/Epoxy Composites and Damage Classification through Principal Component Analysis. Compos. Part B Eng..

[28] Singh S., Kamble Z. (2025). Real-Time Structural Health Monitoring of GFRCs Using RGO-Coated Glass Textile Sensors. Nondestruct. Test. Eval..

[29] del Pilar de Urquijo-Ventura M.P., Rodríguez-González J., Rubio-González C. (2025). Mode I Delamination Monitoring in Carbon Nanotubes-Glass Fiber/Epoxy Composites Using Simultaneous Electrical Self-Sensing and Acoustic Emission Techniques. Compos. Struct..

[30] Bhandari M., Wang J., Jang D., Nam I., Huang B. (2021). A Comparative Study on the Electrical and Piezoresistive Sensing Characteristics of GFRP and CFRP Composites with Hybridized Incorporation of Carbon Nanotubes, Graphenes, Carbon Nanofibers, and Graphite Nanoplatelets. Sensors.

[31] Ku-Herrera J.J., May-Pat A., Avilés F. (2016). An Assessment of the Role of Fiber Coating and Suspending Fluid on the Deposition of Carbon Nanotubes onto Glass Fibers for Multiscale Composites. Adv. Eng. Mater..

[32] Canché H., Oliva-Avilés A.I., Sosa V., Pacheco-Catalán D.E., Cruz-Estrada R.H., Avilés F. (2024). Structure-Property Relationships in the Electrical Conductivity of Multilayer Graphene Sheet/Epoxy Nanocomposites. Diam. Relat. Mater..

[33] (2017). Standard Test Methods for Measuring Adhesion by Tape Test.

[34] (2007). Standard Test Method for Flexural Properties of Polymer Matrix Composite Materials.

[35] Aguilar-Bolados H., Yazdani-Pedram M., Contreras-Cid A., Lopez-Manchado M.A., May-Pat A., Avilés F. (2017). Influence of the Morphology of Carbon Nanostructures on the Piezoresistivity of Hybrid Natural Rubber Nanocomposites. Compos. Part B Eng..

[36] Avilés F., Oliva-Avilés A.I., Cen-Puc M. (2018). Piezoresistivity, Strain, and Damage Self-Sensing of Polymer Composites Filled with Carbon Nanostructures. Adv. Eng. Mater..

[37] Maria M., Navaneethan D., Chan K.H., Taek H.M., Hyun C.K. (2012). Solution Processed Fabrication of Single Wall Carbon Nanotubes Thin Film by Electrohydrodynamic Atomization Deposition Technique and Its Characterization. Appl. Phys. A Mater. Sci. Process..

[38] Castillo J.L., Martin S., Rodriguez-Perez D., Higuera F.J., Garcia-Ybarra P.L. (2018). Nanostructured Porous Coatings via Electrospray Atomization and Deposition of Nanoparticle Suspensions. J. Aerosol Sci..

[39] Park J.S., Kim J.K., Hong J.H., Cho J.S., Park S.K., Kang Y.C. (2019). Advances in the Synthesis and Design of Nanostructured Materials by Aerosol Spray Processes for Efficient Energy Storage. Nanoscale.

[40] Leng J., Wang Z., Wang J., Wu H.H., Yan G., Li X., Guo H., Liu Y., Zhang Q., Guo Z. (2019). Advances in Nanostructures Fabricated: Via Spray Pyrolysis and Their Applications in Energy Storage and Conversion. Chem. Soc. Rev..

[41] Kim G.H., Shin E.A., Jung J.Y., Lee J.Y., Lee C.K. (2022). Effect of Spray Parameters on Electrical Characteristics of Printed Layer by Morphological Study. Processes.

[42] Shou W., Zhang P., Khattab A. (2019). Parametric Study of an Automated Nanoparticles Spray Process for Nanofibers/Fabric Reinforced Composites. Polym. Compos..

[43] Avilés F., May-Pat A., Canché-Escamilla G., Rodríguez-Uicab O., Ku-Herrera J.J., Duarte-Aranda S., Uribe-Calderón J., Gonzalez-Chi P.I., Arronche L., La Saponara V. (2016). Influence of Carbon Nanotube on the Piezoresistive Behavior of Multiwall Carbon Nanotube/Polymer Composites. J. Intell. Mater. Syst. Struct..

[44] Sierra-Chi C.A., Aguilar-Bolados H., López-Manchado M.A., Verdejo R., Cauich-Rodríguez J.V., Avilés F. (2020). Flexural Electromechanical Properties of Multilayer Graphene Sheet/Carbon Nanotube/Vinyl Ester Hybrid Nanocomposites. Compos. Sci. Technol..

[45] Medina C., Balam A., Avilés F. (2024). Thermoresistive Properties of Multilayer Graphene Sheet/Polypropylene Composites Using Four Dissimilar Fillers. Mater. Today Commun..

[46] Li M., Gu Y., Liu Y., Li Y., Zhang Z. (2013). Interfacial Improvement of Carbon Fiber/Epoxy Composites Using a Simple Process for Depositing Commercially Functionalized Carbon Nanotubes on the Fibers. Carbon.

[47] Chuc-Arcia A., Uribe-Riestra G., Avilés F., Rivero-Ayala M.A., Vargas-Coronado R.F., Gutiérrez-Mejía F.A., Perianza-Gómez M., Cauich-Rodríguez J.V. (2026). Enhancing Multilayer Graphene/Glass Fiber Interfacial Bonding and Dispersion through Oxidative Functionalization. Compos. Interfaces.

[48] Jorio A., Dresselhaus M., Saito R., Dresselhaus G.F. (2011). Raman Spectroscopy in Graphene Related Systems.

